# The shared biomarkers and immune landscape in psoriatic arthritis and rheumatoid arthritis: Findings based on bioinformatics, machine learning and single-cell analysis

**DOI:** 10.1371/journal.pone.0313344

**Published:** 2024-11-07

**Authors:** Kaiyi Zhou, Siyu Luo, Qinxiao Wang, Sheng Fang

**Affiliations:** Department of Dermatology, The First Affiliated Hospital of Chongqing Medical University, Chongqing, China; Sichuan University, CHINA

## Abstract

**Objective:**

Psoriatic arthritis (PsA) and rheumatoid arthritis (RA) are the most common types of inflammatory musculoskeletal disorders that share overlapping clinical features and complications. The aim of this study was to identify shared marker genes and mechanistic similarities between PsA and RA.

**Methods:**

We utilized datasets from the Gene Expression Omnibus (GEO) database to identify differentially expressed genes (DEGs) and perform functional enrichment analyses. To identify the marker genes, we applied two machine learning algorithms: the least absolute shrinkage and selection operator (LASSO) and the support vector machine recursive feature elimination (SVM-RFE). Subsequently, we assessed the diagnostic capacity of the identified marker genes using the receiver operating characteristic (ROC) curve and decision curve analysis (DCA). A transcription factor (TF) network was constructed using data from JASPAR, HumanTFDB, and GTRD. We then employed CIBERSORT to analyze the abundance of immune infiltrates in PsA and RA, assessing the relationship between marker genes and immune cells. Additionally, cellular subpopulations were identified by analyzing single-cell sequencing data from RA, with T cells examined for trajectory and cellular communication using Monocle and CellChat, thereby exploring their linkage to marker genes.

**Results:**

A total of seven overlapping DEGs were identified between PsA and RA. Gene enrichment analysis revealed that these genes were associated with mitochondrial respiratory chain complex IV, Toll-like receptors, and NF-κB signaling pathways. Both machine learning algorithms identified Ribosomal Protein L22-like 1 (RPL22L1) and Lymphocyte Antigen 96 (LY96) as potential diagnostic markers for PsA and RA. These markers were validated using test sets and experimental approaches. Furthermore, GSEA analysis indicated that gap junctions may play a crucial role in the pathogenesis of both conditions. The TF network suggested a potential association between marker genes and core enrichment genes related to gap junctions. The application of CIBERSORT and single-cell RNA sequencing provided a comprehensive understanding of the role of marker genes in immune cell function. Our results indicated that RPL22L1 and LY96 are involved in T cell development and are associated with T cell communication with NK cells and monocytes. Notably, high expression of both RPL22L1 and LY96 was linked to enhanced VEGF signaling in T cells.

**Conclusion:**

Our study identified RPL22L1 and LY96 as key biomarkers for PsA and RA. Further investigations demonstrated that these two marker genes are closely associated with gap junction function, T cell infiltration, differentiation, and VEGF signaling. Collectively, these findings provide new insights into the diagnosis and treatment of PsA and RA.

## 1. Introduction

PsA is a chronic, inflammatory, and heterogeneous arthropathy characterized by immune cell infiltration of the synovium and increased angiogenesis. The estimated prevalence of PsA is 0.3% to 1% and affects approximately 20% to 30% of patients with cutaneous psoriasis [1–3]. Since PsA exhibits seronegativity, diagnosis relies on the identification of clinical and imaging characteristics, such as skin psoriasis, nail psoriasis, peripheral joint disease, axial skeleton involvement, enthesitis, and dactylitis [4]. Rheumatoid arthritis (RA) is another prevalent inflammatory musculoskeletal disease that leads to synovial inflammation and osteochondral damage, with an incidence of 0.5% to 1% [5]. Unlike PsA, RA autoantibodies, including rheumatoid factor and anti-cyclic citrullinated peptide antibodies, can be detected in the bloodstream during the pre-articular phase of RA [6]. Joint involvement in RA can overlap with PsA, characterized by symmetrical distribution, multiple joint involvement, peripheral joint destruction, and spinal involvement. The symptoms and signs of RA and PsA are often similar, particularly during the early stages of the conditions [4, 7].

Immune cells in the peripheral blood release pro-inflammatory mediators that abnormally regulate synovial resident cells, resulting in the destruction of articular cartilage and bone, which ultimately leads to PsA [8]. The progression of RA occurs in three distinct stages: autoantibody production, acute arthritis, and chronic arthritis. During these stages, the immune system remodels, leading to weakened tissue tolerance and the generation of autoaggressive effector cells [9]. Immune-mediated rheumatic diseases extend beyond arthritic manifestations, causing pathological changes in various organs through the bloodstream. The term psoriatic disease emphasizes a systemic condition encompassing PsA, extra-articular symptoms (such as psoriasis, inflammatory bowel disease, and uveitis), and complications (such as cardiovascular disease, metabolic syndrome, and mental disorders) [10, 11]. RA is also recognized as a syndrome with additional extra-articular manifestations, including cutaneous manifestations and vasculitis, along with systemic comorbidities, such as cardiovascular, pulmonary, and neurological issues [12, 13]. Given the systemic and multi-organ nature of PsA and RA, identifying blood markers and exploring the common pathogenesis in terms of immunity is critical. We summarised the common biomarkers for PSA and RA based on clinical trial reports (**Table 1**). As demonstrated, the current research is far from adequate.

**Table 1:** Shared biomarkers between psoriatic arthritis and rheumatoid arthritis from clinical trials.

Biomarker	Tissue	Year	Reference
A-SAA	peripheral blood	2000	[14]
S100A12	synovial	2003	[15]
VEGF, bFGF	synovial fluid, peripheral blood	2004	[16]
PRL	synovial fluid, peripheral blood	2017	[17]
IL-19, IL-20, IL-24	synovial fluid, peripheral blood	2015	[18]

The present study employed bioinformatics analysis and machine learning methods to identify common marker genes for PsA and RA. The study also investigated immune infiltration using CIBERSORT and single-cell analysis. Additionally, the relationships between marker genes and immune cells were explored to provide deeper insights into the molecular immune mechanisms underlying PsA and RA.

## 2. Materials and methods

### 2.1. Data collection and preprocessing

**Fig 1:**
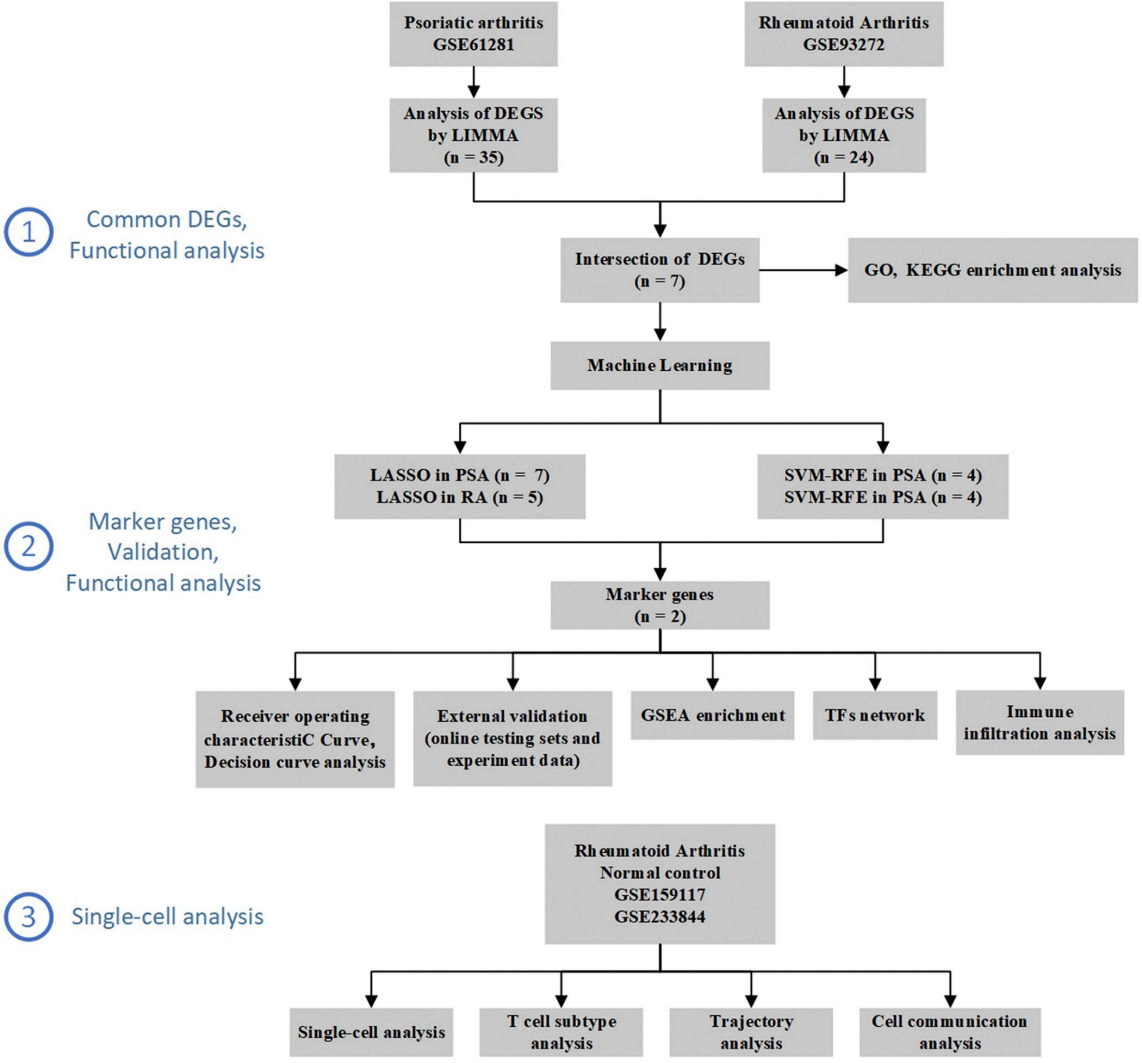
The workflow of the present study.

**Fig 1** illustrates a workflow of this study. All datasets were retrieved from the GEO database (http://www.ncbi.nlm.nih.gov/geo/) in May 2023. The inclusion criteria were as follows: (1) Tissue samples were derived from human patients, not animals or cell lines; (2) Patients had not undergone any specific treatments; and (3) Normal control samples were available. Ultimately, six human microarray datasets were selected. Two training sets, GSE61281-GPL6480 and GSE93272-GPL570, were obtained from whole blood samples of patients with psoriatic arthritis (PsA) and rheumatoid arthritis (RA), respectively. Four test sets—GSE13355, GSE15573, GSE77298, and GSE82107—were sourced from various tissues, including peripheral blood mononuclear cells, synovium, and skin. Further details regarding the selected datasets are provided in **Table 2**.

**Table 2:** The information of datasets used in this study.

GSE number	Platform	Sequencing type	Patient	Healthy control	Disease	Tissue	Type
GSE61281	GPL6480	Bulk-seq	20	12	PsA	PBMC	Training
GSE93272	GPL570	Bulk-seq	232	43	RA	PBMC	Training
GSE13355	GPL570	Bulk-seq	58	64	PsA	Skin	Test
GSE15573	GPL6102	Bulk-seq	18	15	RA	PBMC	Test
GSE77298	GPL570	Bulk-seq	26	14	RA	Synovium	Test
GSE82107	GPL570	Bulk-seq					
GSE159117	GPL23227	scRNA-seq	1	0	RA	PBMC	Test
GSE233844	GPL20301	scRNA-seq	0	3	Normal	PBMC	Test

For GSE61281, we utilized 20 samples from individuals with psoriatic arthritis (GSM1501512–GSM1501531) and 12 samples from healthy controls (GSM1501552 –GSM1501563). We combined datasets GSE77298 and GSE82107, and eliminated any batch effects using the ComBat algorithm from the R package “sva” (version 3.46.0, https://bioconductor.org/packages/release/bioc/html/sva.html) [19]. Background correction and normalization of all datasets were performed using the “limma” package (version 3.54.0) in R, and for genes with multiple probes, the mean probe value was used for ID transformation.

### 2.2. Screening of DEGs

Differential gene expression in the training sets GSE61281 and GSE93272 was analyzed using the "limma" R package. Genes with an adjusted *p*-value < 0.05 and |fold change (FC)| > 2 were considered significant. The identified DEGs were visualized through volcano plots and heat maps, generated using the "ggplot2" (version 3.4.1) and "pheatmap" (version 1.0.12) R packages, respectively. Additionally, a Venn diagram, created with the "ggVennDiagram" (version 1.2.2) package, illustrated the intersection of the DEGs under investigation.

### 2.3. Identification for marker genes based on two machine learning algorithms

The least absolute shrinkage and selection operator (LASSO) is a machine learning regression model that uses L1-regularization to prevent overfitting [20]. In machine learning and pattern recognition, Support Vector Machines (SVMs) are widely used due to their ability to minimize both structural risk and empirical error, thereby improving learning performance. SVM-RFE is an emerging bio-information mining technique that uses a defined training model and cross-validation to obtain the feature gene with the least error. LASSO emphasizes sparsity and regularization, while SVM-RFE focuses on classification accuracy and feature ranking. Using both ensures that the selected biomarkers are not only statistically significant but also relevant for classification.

In this study, we employed the "glmnet" (version 4.1–6) and "e1071" (version 1.7–13) R packages to perform LASSO and SVM-RFE analyses on seven overlapping DEGs to identify marker genes for PsA and RA. The response type for LASSO was set as binomial, and alpha was set as 1. SVM-RFE was configured with k = 10 for k-fold cross-validation, and a halving parameter of 10 was used. The intersection of the results from both algorithms was considered the set of marker genes.

### 2.4. Clinical application analysis and verification of marker genes

To evaluate the discriminatory ability and clinical utility of the identified marker genes, we generated ROC curves and DCA using the R packages "pROC" (version 1.18.0) and "ggDCA" (version 1.2), respectively, based on data from the two test sets, GSE61281 and GSE93272. For validation, we used four independent datasets—GSE13355, GSE15573, GSE77298, and GSE82107—and visualized gene expression via box plots. Statistical significance was assessed using t-tests. Additionally, ROC curves were plotted using data from the four validation sets and quantitative RT-PCR results.

### 2.5. Functional and pathway enrichment analysis

Gene Ontology (GO) and Kyoto Encyclopedia of Genes and Genomes (KEGG) analyses were conducted on seven DEGs using the R package “clusterProfiler”. Unlike KEGG analysis, which focuses on a subset of genes, Gene Set Enrichment Analysis (GSEA) examines the distribution of predefined gene sets within the entire ranked gene table, enabling assessment of the contribution of genes to enriched pathways. Genes that significantly contribute to the enrichment score are termed core enrichment genes. In our study, GSEA was performed to evaluate the signaling pathways associated with two marker genes. Samples were classified into high and low expression groups based on the median expression values of the marker genes. DEGs between the two groups were calculated using the "limma" package, and all genes were ranked according to their log2 fold change (log2FC) values. MSigDB (https://www.gsea-msigdb.org/gsea/msigdb/index.jsp) was chosen as the reference gene set [21]. A gene set and pathways was regarded as significantly enriched when *p* < 0.05.

### 2.6. TF network of two marker genes and core enrichment genes

We identified the transcription factors (TFs) associated with the ten core enrichment genes using three transcription factor databases: JASPAR, HumanTFDB, and GTRD. The overlapping results from these databases were considered the final set of TFs. The regulatory network of the TFs was visualized using Cytoscape (version 3.9.1, https://cytoscape.org/), and node sizes were calculated using the Betweenness method with the built-in CytoNCA plugin [22, 23].

### 2.7. Immune infiltration analysis

The bioinformatics algorithm CIBERSORT (https://cibersortx.stanford.edu/) was utilized to calculate the abundance of 22 types of immune cell infiltration [24]. Our study quantified the relative proportions of infiltrating immune cells in PsA, rheumatoid arthritis RA, and their respective control groups. The correlation between marker genes and 22 immune-infiltrated cells was determined using Spearman’s correlation analysis. R packages “ggplot2” and “corrplot” (version 0.92) were employed to illustrate the results of the correlation analysis.

### 2.8. Single-cell RNA-sequencing data

The scRNA-seq datasets GSE176078 and GSE233844 were downloaded from the GEO database. One sample of RA peripheral blood mononuclear cells (PBMCs) and three normal control samples were included in the analysis. Quality control procedure was performed to filter out single cells: 1) each gene expressed more than 300 genes and was expressed in more than five cells; 2) the number of expressed genes in cells was between 200 and 4500; 3) the percentage of mitochondrial genes in a cell was less than 10%; 4) the number of unique molecular identifiers (UMI) was more than 1000. Then, R software and Seurat package (version 4.4.1) were used for data processing, scaling, dimension reduction and cell clustering. The Harmony package was used to eliminate batch effects between samples. Subsequently, “FindClusters” and “FindNeighbors” functions were conducted to cluster single cells into different subgroups under conditions of dim = 25 and resolution = 0.8. Finally, single cells were visualized using the Uniform Manifold Approximation and Projection (UMAP) dimensionality reduction technique. Manual annotation was performed for cell identification [25, 26].

The Monocle package (version 2.32.0) was used to analyze the pseudo-time trajectories of T cells. We used “DDRTree” function and a setting of max components  =  2 parameters to downscale cell data. The visualization of cell branching and marker gene expression trends was conducted using the functions "plot_cell_trajectory" and "plot_pseudotime_heatmap".

The R package CellChat enables the inference, visualization, and analysis of cell-cell communication on scRNA-seq data [27]. To investigate T cells communication and its relationship with marker genes, we used the median value of marker genes expression to classify T cells into high and low expression groups. Subsequently, the ligand-receptor interactions between different cells were analyzed using the package Cellchat (version 1.6.1).

### 2.9. Quantitative RT-PCR

Archived whole blood samples were used in this study from patients who presented to the First Hospital of Chongqing Medical University from August 2023 to April 2024. Three PsA patients (aged 44–65 years), three RA patients (aged 48–67 years), and three healthy controls (aged 45–55 years) were included. All patients presented with at least one extra-articular complication. The data were accessed in April 2024. Our study was approved by the Ethics Committee of the First Affiliated Hospital of Chongqing Medical University. The Ethics Committee waived the requirement for consent because the samples used were collected from previous consultations. The authors had access to information that could identify individual participants during and after data collection. Blood RNA Extraction Kit (Accurate Biotechnology, China) was used to extract RNA from blood sample. Process 1.5 mL blood samples individually according to the instructions provided and use Nanodrop to measure concentration and purity. RT Kit with gDNA Clean (Accurate Biotechnology, China) was used to remove genomic DNA and obtain cDNA. No more than 1μg of total RNA should be used in each addition. With the SYBR Premix Reagent (TianGen Biotech, China), RT-qPCR proceeded in the Bio-RadCFX Duet system. The primer of different genes needed in our research is shown in the **S1 Table**. Target genes were normalized to GAPDH using the comparative Cq method.

## 3. Results

### 3.1. Identification of DEGs in PsA and RA

**Fig 2:**
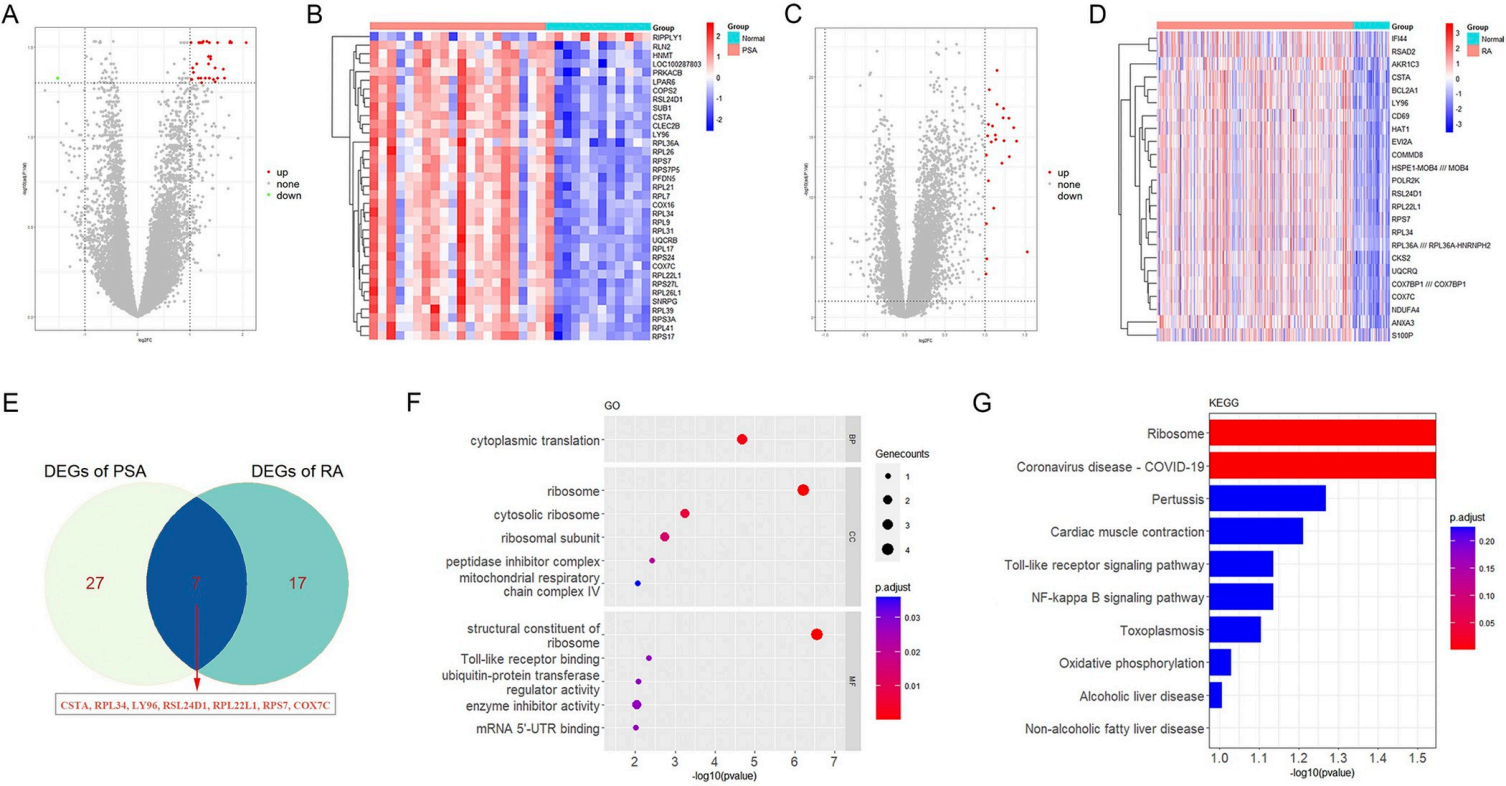
Differentially expressed genes and their enrichment analysis in PsA and RA training cohort. (A) Volcano plot providing visual representation of the significance and fold-change of differentially expressed genes in GSE61281 (PsA). (B) Heatmap displaying expression levels of DEGs in GSE61281 (PsA). (C) Volcano plot displaying DEGs in GSE93272 (RA). (D) Heatmap of DEGs in GSE93272 (RA). (E) Venn plot showing the intersection of DEGs of two diseases. F) Bubble diagram shows the significant GO terms for 7 DEGs. (G) Bar graph shows the significant pathways for 7 DEGs.

In the PsA dataset GSE61281, a total of 35 DEGs were identified when compared to healthy controls. Among these, 34 genes were upregulated, while 1 gene was downregulated, as illustrated in the volcano plot and heatmap (**Fig 2A and 2B**). In the RA dataset GSE93272, 24 DEGs were screened, all of which were upregulated when compared to healthy controls, as depicted in the volcano plot and heatmap (**Fig 2C and 2D**).

### 3.2. Enrichment analysis based on DEGs

As shown in **Fig 2E**, a total of 7 overlapping elements were identified as shared differentially expressed genes (DEGs) between PsA and RA. **Fig 2F** presents the top annotations from Gene Ontology (GO) analysis, which encompass four key domains: (I) ribosome and translation processes, including cytoplasmic translation and ribosomal functions; (II) regulation of enzyme activity; (III) mitochondrial respiratory chain complexes; and (IV) Toll-like receptor binding. The KEGG analysis highlights three highly enriched pathways: (I) the immune-related NF-κB and Toll-like receptor signaling pathways; (II) complications and associated pathways, such as oxidative phosphorylation, non-alcoholic fatty liver disease, alcoholic liver disease, and cardiac muscle contraction; and (III) infectious diseases, including COVID-19, pertussis, and toxoplasmosis (**Fig 2G**).

### 3.3. Identification of marker genes

**Fig 3:**
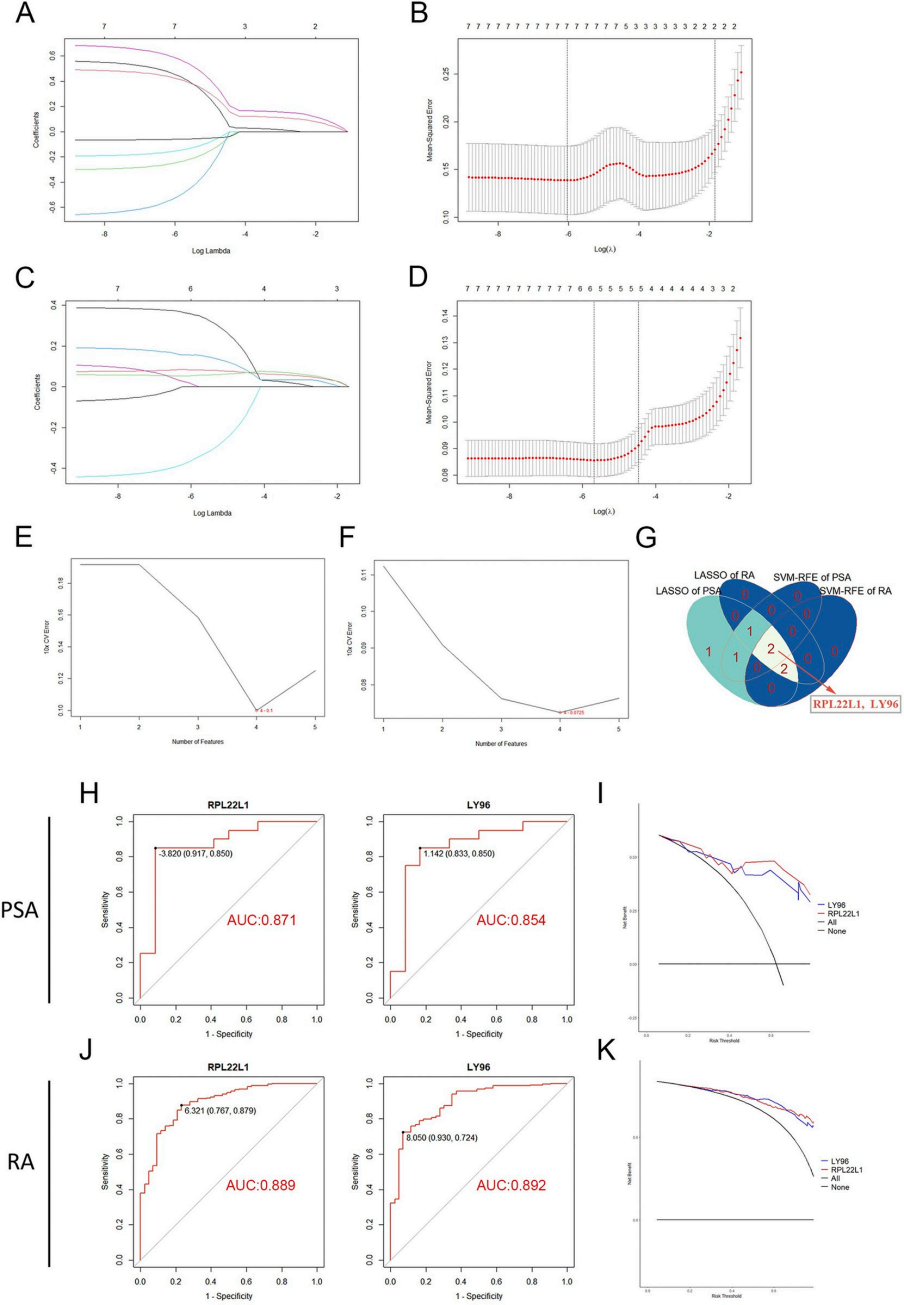
Screening process for marker genes from 7 DEGs and the diagnostic value of marker genes. (A-D) The LASSO regression algorithm to screen candidate marker genes in PsA dataset GSE61281 (A, B) and RA dataset GSE93272 (C, D). (E, F) The SVM-RFE algorithm to screen candidate marker genes in PsA dataset GSE61281 (C) and RA dataset GSE93272 (D). (G) The Venn diagram highlights the intersection of candidate marker genes in two diseases and two algorithms. (H, J) ROC curves assess the diagnostic ability of marker genes in the training cohort. (I, K) DCA curves evaluate the practical value of marker genes in the training cohort.

To identify potential marker genes from the 7 overlapping DEGs, two machine learning algorithms were employed. The LASSO logistic regression algorithm identified 7 crucial genes in PsA and 5 genes (COX7C, CSTA, LY96, RPL22L1, and RPL34) in RA (**Fig 3A–3D**). Additionally, the SVM-RFE algorithm identified 4 specific genes (RPS7, CSTA, RPL22L1, and LY96) in the PsA cohort, as well as 4 genes (COX7C, RPL34, RPL22L1, and LY96) in the RA cohort (**Fig 3E and 3F**). Ultimately, by combining the results from both algorithms, we identified two shared marker genes (RPL22L1 and LY96) for PsA and RA (**Fig 3G**).

### 3.4. Diagnostic value evaluation

To assess the diagnostic utility of RPL22L1 and LY96, ROC assays were performed using the training sets GSE61281 and GSE93272. The results, illustrated in **Fig 3H and 3J**, demonstrate the strong diagnostic performance of RPL22L1 and LY96 in differentiating disease samples from healthy samples, with all AUC > 0.85. Additionally, the practical value of RPL22L1 and LY96 was evaluated using DCA curves (**Fig 3I and 3K**). Within risk thresholds ranging from 0 to 0.75, the curves for RPL22L1 and LY96 consistently surpassed both the "all" and "none" curves, suggesting that decision-making based on these marker genes may provide significant benefits for patients with PsA and RA.

### 3.5. Verification of marker genes based on test sets and experiment data

**Fig 4:**
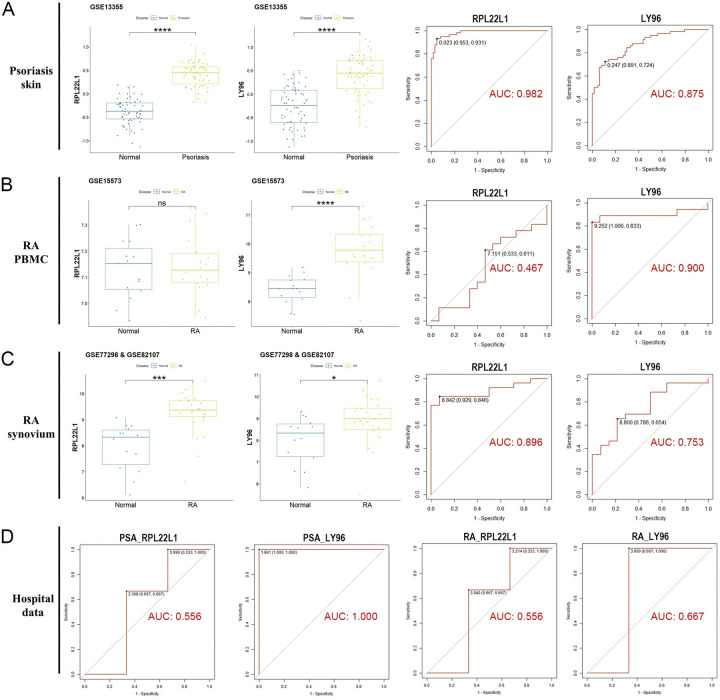
Verification of marker genes. (A, B, C) Box plots and ROC curves assess the differential expression and diagnostic ability of marker genes in psoriatic skin (A), RA peripheral blood mononuclear cells (B), and RA synovial fluid (C). (D) ROC curves assess the diagnostic ability of marker genes in whole blood experimental data. ns *p* > 0.05, * *p* < 0.05, *** *p* < 0.001, **** *p* < 0.0001.

To further validate the critical role of the two marker genes, external verification was conducted. Due to the absence of PsA blood sequencing data in existing databases, we selected psoriasis skin biopsy microarray data from GEO to assess the expression differences of the marker genes. For RA, both blood and synovium biopsy microarray data were utilized. **Fig 4A–4C** illustrate that, in the majority of datasets, RPL22L1 and LY96 exhibit significant expression variation between diseased and normal samples, demonstrating strong diagnostic potential. However, RPL22L1 did not yield significant results in RA PBMC samples (*p* > 0.05, AUC = 0.467). Experimental data from our hospital further confirmed the diagnostic efficacy of LY96 in both diseases, while RPL22L1 showed limited effectiveness (**Fig 4D**).

### 3.6. Enrichment analysis based on marker genes

**Fig 5:**
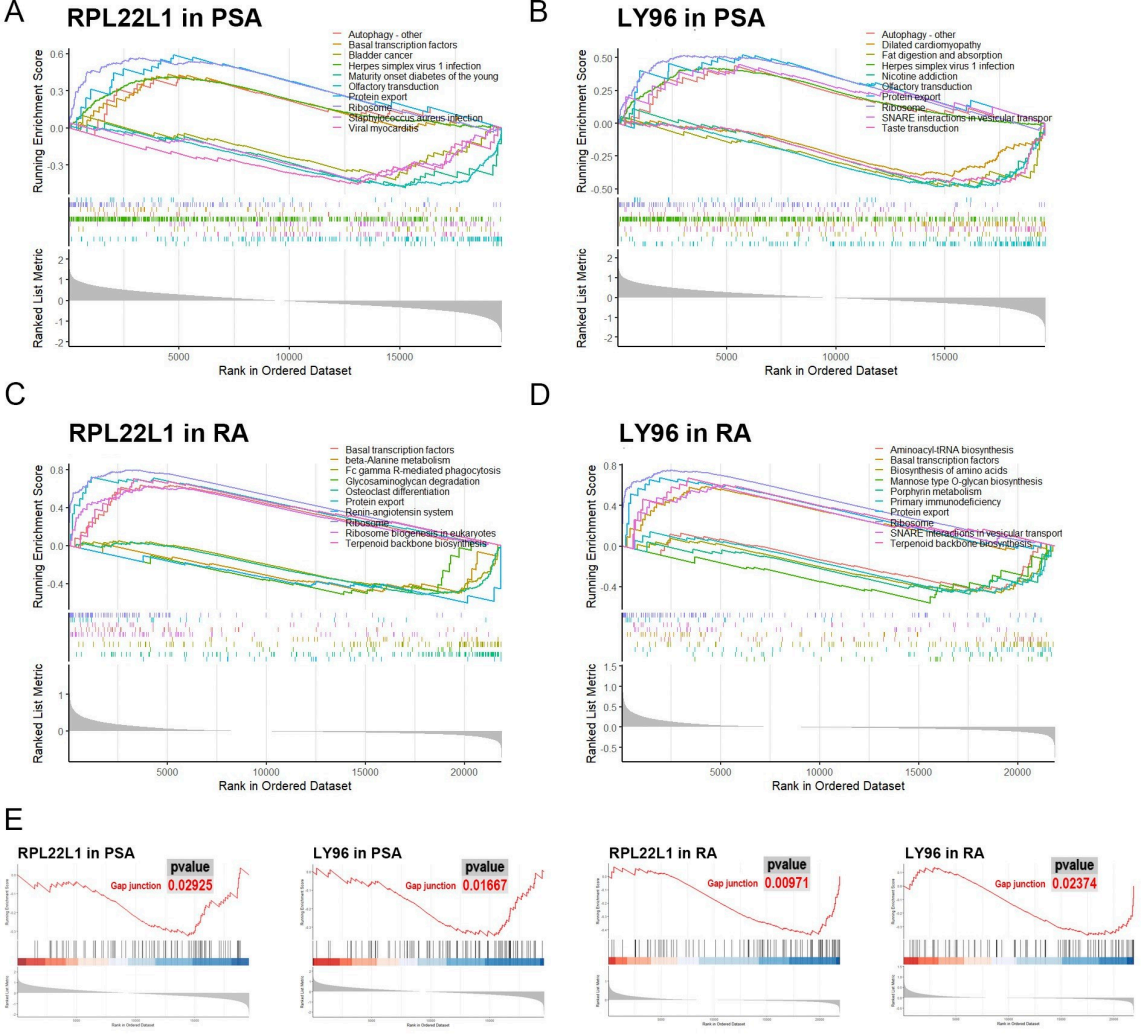
Gene set enrichment analysis. (A) Top ten significantly enriched pathways of RPL22L1 in PsA dataset. (B) Top ten significantly enriched pathways of LY96 in PsA dataset. (C) Top ten significantly enriched pathways of RPL22L1 in RA dataset. (D) Top ten significantly enriched pathways of LY96 in RA dataset. (E) The results of GSEA demonstrate significant enrichment in the gap junction pathway.

To identify potential pathways involving the marker genes, we conducted GSEA. The results highlighted pathways related to material metabolism, infectious diseases, and those directly associated with PsA and RA, including osteoclast differentiation, autophagy, SNARE interactions in vesicular trafficking, and Fc gamma receptor-mediated phagocytosis (**Fig 5A–5D**). Furthermore, we identified significant pathways related to intercellular connectivity, such as gap junctions, tight junctions, focal adhesions, and adherens junctions (**S2 Table**). To explore the shared pathways influenced by RPL22L1 and LY96 in both PsA and RA, we examined the pathways enriched by these genes and identified nine outcomes (**S1 Fig and S3 Table**). Notably, gap junctions emerged as a point of particular interest (**Fig 5E**).

### 3.7. Transcription factors of marker genes and gap junction core enrichment genes

**Fig 6:**
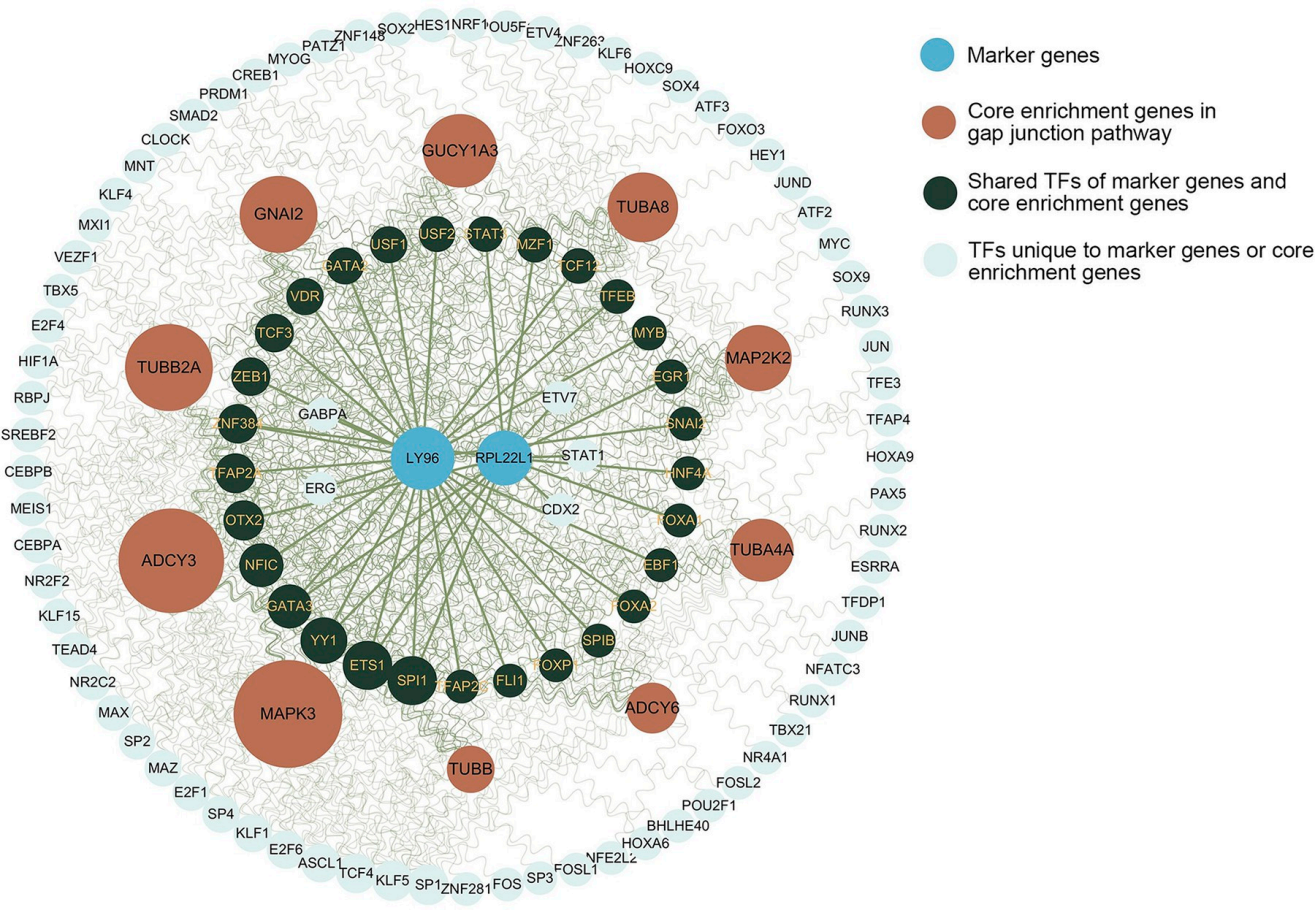
TF network. The size of the circle is proportional to its betweenness centrality. Brown circles represent 2 marker genes and 10 core enrichment genes in gap junction pathway. The bottle-green shows mutual TFs of marker genes and core enrichment genes. The pale blue denotes TFs unique to marker genes or core enrichment genes.

To investigate the latent correlation between the two marker genes and gap junctions, we identified core enrichment genes within the gap junction pathway and found an overlap of 10 genes (**Fig 6**). We retrieved 102 transcription factors associated with these 10 genes from three prediction databases, with their regulatory network illustrated in **Fig 6**. Notably, 29 TFs were found to modulate both the marker genes and the core enrichment genes related to gap junctions, of which five TFs (SPI1, ETS1, YY1, GATA3, ZNF384) influence both LY96 and RPL22L1.

### 3.8. Abundance of immune cells

**Fig 7:**
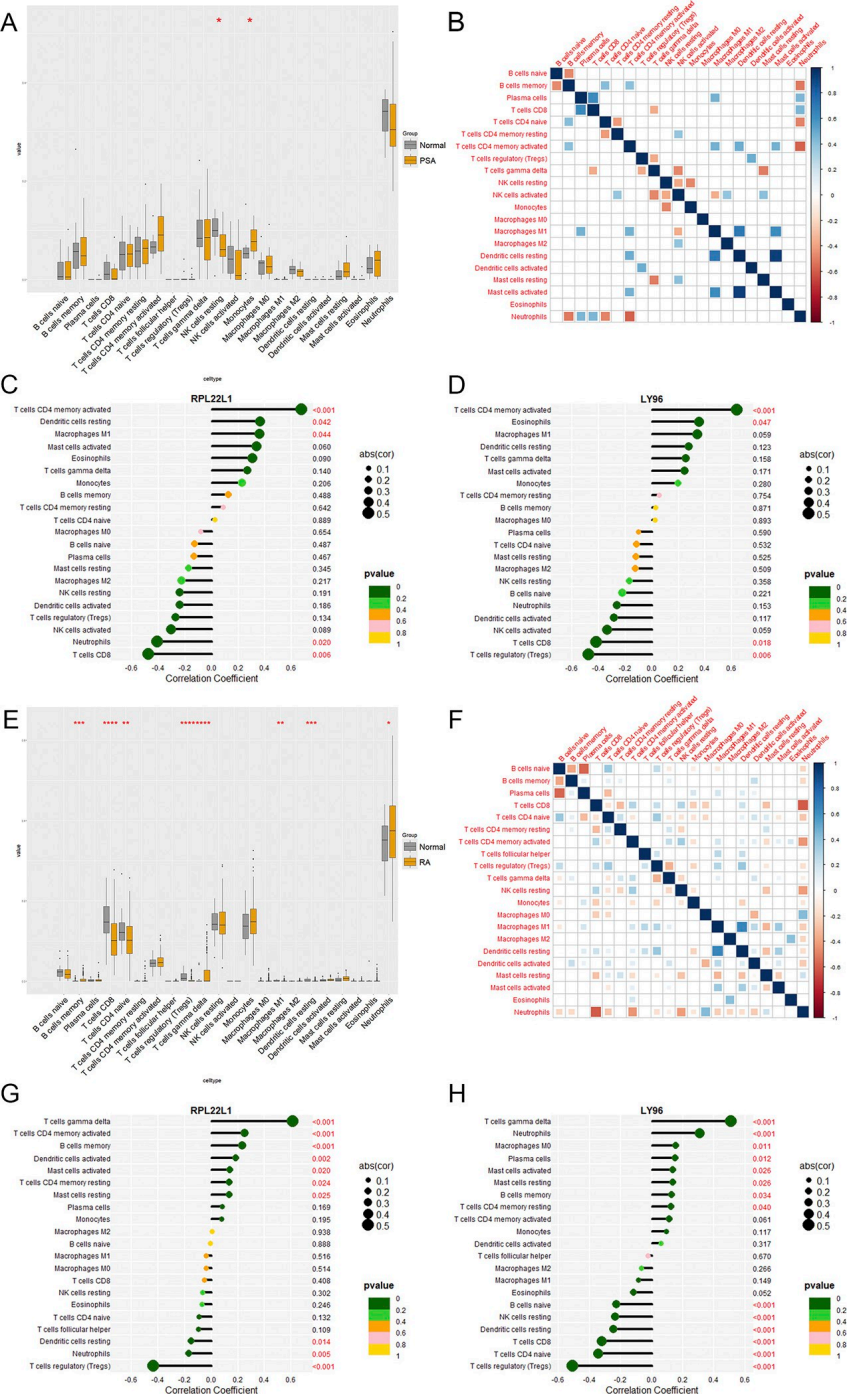
Immune infiltration analysis of PsA and RA. (A, E) Boxplot of the proportions of 22 infiltrating immune cells in PsA(A) and RA (E). (B, F) Correlation of 22 immune cell type compositions in PsA (B) and RA (F). (C) Correlation between RPL22L1 and infiltrating immune cells in PsA. (D) Correlation between LY96 and infiltrating immune cells in PsA. (G) Correlation between RPL22L1 and infiltrating immune cells in RA. (H) Correlation between RPL22L1 and infiltrating immune cells in RA.

Immune dysfunction contributes to the development of both PsA and RA, as supported by our function enrichment findings. We conducted an immune cell infiltration analysis using gene expression data from GSE61281 and GSE93272. In GSE61281, we observed significantly higher levels of monocytes (*p* < 0.005) and lower levels of resting NK cells (*p* < 0.005) in the PsA group compared to the control group (**Fig 7A**). The correlation of 22 types of immune cells revealed that activated mast cells were positively associated with resting dendritic cells (r = 0.93) and that M1 macrophages were positively related to resting dendritic cells (r = 0.70), whereas neutrophils were negatively related to activated CD4 memory T cells (r = −0.59; >**Fig 7B**). As illustrated in **Fig 7C**, RPL22L1 was positively correlated with activated memory CD4 T cells (r = 0.67, *p* < 0.01), resting dendritic cells (r = 0.36, *p* < 0.05) and M1 macrophages (r = 0.36, *p* < 0.05) and negatively correlated with neutrophils (r = -0.41, *p* < 0.05) and CD8 T cells (r = -0.47, *p* < 0.01). LY96 exhibited positive correlations with activated memory CD4 T cells (r = 0.64, *p* < 0.001) and eosinophils (r = 0.35, *p* < 0.05), while presenting negative correlations with CD8 T cells (r = -0.42, *p* < 0.05) and regulatory T cells (r = -0.47, *p* < 0.01; **Fig 7D**). In GSE93272, the results showed that the proportions of memory B cells (*p* < 0.001), gamma delta T cells (*p* < 0.0001), and neutrophils (*p* < 0.05) were significantly higher in the RA group than in the normal group. However, CD8 T cells (*p* < 0.0001), naïve CD4 T cells (*p* < 0.01), regulatory T cells (*p* < 0.0001), M1 macrophages (*p* < 0.01), and resting dendritic cells (*p* < 0.001) were significantly lower in the RA group than in the normal group (**Fig 7E**). The correlation of 22 types of immune cells revealed that resting dendritic cells were positively associated with M1 macrophages (r = 0.64), whereas neutrophils were negatively related to CD8 T cells (r = −0.60; **Fig 7F**). As demonstrated in **Fig 7G**, RPL22L1 exhibited a significant positive correlation with gamma delta T cells (r = 0.61, *p* < 0.001), activated memory CD4 T cells (r = 0.25, *p* < 0.001), memory B cells (r = 0.23, *p* < 0.001), activated dendritic cells (r = 0.19, *p* < 0.01), activated mast cells (r = 0.14, *p* < 0.05), resting memory CD4 T cells (r = 0.14, *p* < 0.05) and resting mast cells (r = 0.14, *p* < 0.05), while negatively correlated with resting dendritic cells (r = -0.15, *p* < 0.05), neutrophils (r = -0.17, *p* < 0.001) and regulatory T cells (r = -0.43, *p* < 0.001). LY96 was positively correlated with gamma delta T cells (r = 0.51, *p* < 0.001), neutrophils (r = 0.31, *p* < 0.001), M0 macrophages (r = 0.15, *p* < 0.05), plasma cells (r = 0.15, *p* < 0.05), activated mast cells (r = 0.13, *p* < 0.05), resting mast cells (r = 0.13, *p* < 0.05), memory B cells (r = 0.13, *p* < 0.05) and resting memory CD4 T cells (r = 0.12, *p* < 0.05), while negatively correlated with naïve B cells (r = -0.23, *p* < 0.001), resting NK cells (r = -0.23, *p* < 0.001), resting dendritic cells (r = -0.25, *p* < 0.001), CD8 T cells (r = -0.32, *p* < 0.001), naïve CD4 T cells (r = -0.35, *p* < 0.001), regulatory T cells (r = -0.51, *p* < 0.001; **Fig 7H**).

### 3.9. Annotating cell types in single-cell data of RA patients

**Fig 8:**
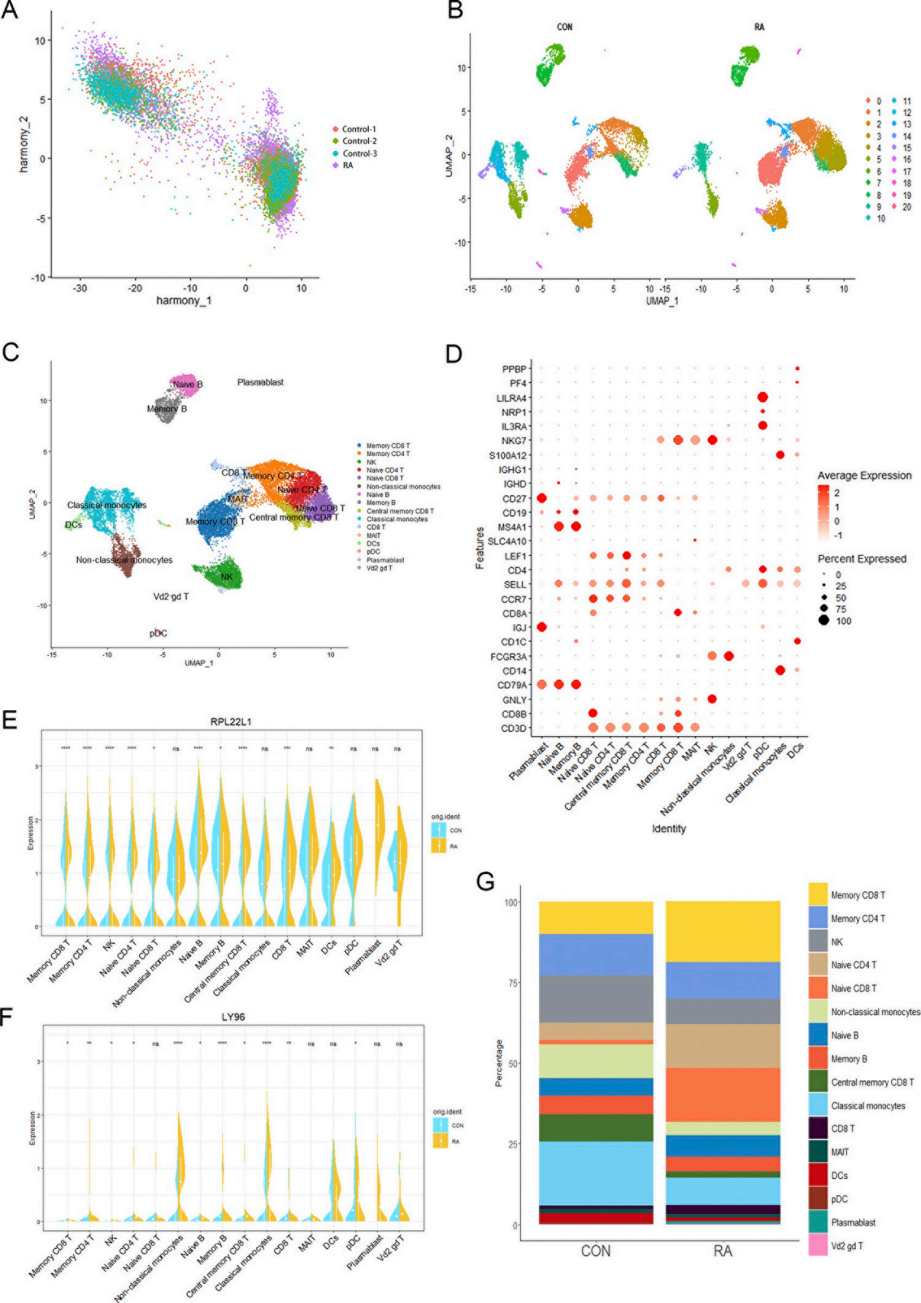
Single-cell sequencing analysis. (A) Harmony plot colored by one RA and three normal samples. (B) UMAP plot colored by various cell clusters. (C) UMAP plot colored by cells after annotation. (D) Heat map shows the expression of hallmark genes in different cell clusters from RA PBMCs. The scaled average expression levels of marker genes and the percentage of cells expressing marker genes are expressed by color and size of each dot corresponding to cell clusters, respectively. (E, F) The violin plots illustrate the expression of RPL22L1 (E) and LY96 (F) in each of the cell clusters within the RA and control groups. (G) Stacked bar plot to demonstrate cell percentage between control and RA samples.

To characterize the changes of gene expression in the peripheral blood of RA patients, we obtained single-cell RNA sequencing data from GSE159117 and GSE233844 in the GEO database, which included one RA patient and three normal controls. After filtering out low-quality cells, normalizing, integrating, and performing principal component analysis (PCA) and batch removal using Harmony, we identified 19,167 cells grouped into 20 clusters (**Fig 8A and 8B**). Further, according to the markers of peripheral blood mononuclear cells (**Fig 8D**), we identified 16 cell types, including memory CD8+ T cells, memory CD4+ T cells, NK cells, naïve CD4+ T cells, naïve CD8+ T cells, non-classical monocytes, naïve B cells, memory B cells, Central memory CD8+ T cells, classical monocytes, CD8+ T cells, MAIT cells, Dendritic cells, Plasmacytoid dendritic cells, plasmablast and Vd2 gd T cells (**Fig 8C**). Notably, the proportions of memory CD8+ T cells, naïve CD4+ T cells, and naïve CD8+ T cells were higher in RA patients compared to normal controls (**Fig 8G**). Additionally, we utilized violin plots to visualize the expression levels of the two marker genes across different cell types. Significant differences in the expression of RPL22L1 and LY96 were observed in T cell subsets, B cell subsets, and NK cells in RA patients compared to the normal group (**Fig 8E and 8F**).

### 3.10. Trajectory analysis

**Fig 9:**
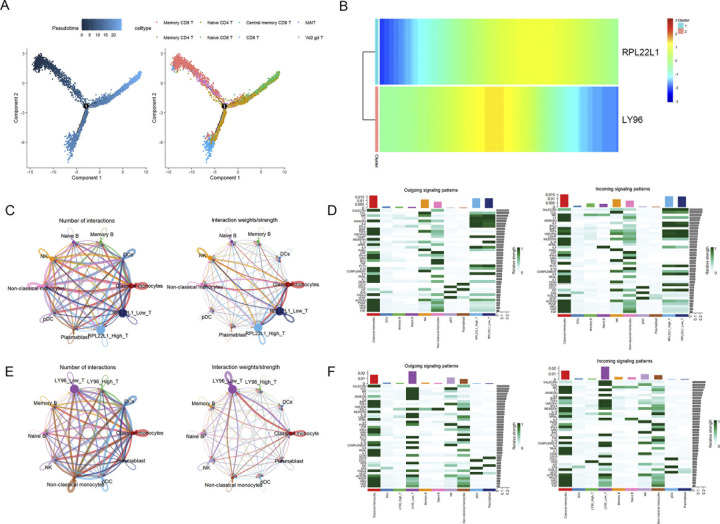
Trajectory and cell-cell communication analysis. (A) Differentiation trajectory results for T cells. (B) Heatmap of marker genes expression along the pseudotime trajectory. (C, E) The number and weight of interaction in cell-cell communication network. (D, F) Heatmap visualizing the possible incoming or outgoing signaling pathways among cell.

Given that both the CIBERSORT immune infiltration analysis and single-cell data indicated potential involvement of the marker genes in T cells, we performed a pseudotime analysis of this cell type. As shown in **Fig 9A**, T cells can be broadly categorized into three differentiation states. Early naïve cells differentiate into functional T cells, while memory cells are observed at all stages. The heatmap representing cellular development revealed that RPL22L1 is expressed during the middle to late stages of differentiation, whereas LY96 is predominantly expressed in the middle stage (**Fig 9B**).

### 3.11. Cell-cell interaction analysis

To gain further insight into the role of the marker genes in T cell communication, T cells in the RA sample were categorized into high and low expression groups based on the median expression levels of the marker genes. The results indicated that T cells interacted closely with NK cells and monocytes. T cells in the RPL22L1 high expression group sent and received more signals overall, while VEGF signal reception was lost in the RPL22L1 low expression group (**Fig 9C and 9D**). In contrast, T cells communication was significantly impaired in the LY96 high expression group, affecting signals including CCL, IL2, LT, MIF, TGFb, PARs, FLT3, IL6, CD40, FASLG, ACTIVIN, FGF, with the exception of VEGF signalling (**Fig 9E and 9F**). We have summarized the key findings of this study in **Table 3**.

**Table 3:** 

Shared DEGs^1^	GO analysis based on shared DEGs^1^	KEGG analysis based on shared DEGs^1^	Marker genes based on LASSO and SVM-RFE^1^	Shared GSEA pathways based on marker genes^1^	TFs shared by marker genes and gap junction core enrichment genes^1^	Shared immune infiltration cell types association with RPL22L1 using CIBERSORT^1^	Shared immune infiltration cell types association with LY96 using CIBERSORT^1^	Immune infiltration cell types association with both RPL22L1 and LY96 in RA using scRNA-seq analysis^2^	Expression stages of marker genes during T cell differentiation^2^	T cell communication signaling association with both RPL22L1 and LY96 in RA^2^
CSTA	Ribosome and translation processes	NF-κB signaling pathways	RPL22L1	Coronavirus disease—COVID-19	SPI1	Activated memory CD4 T cells	CD8 T cells	T cell subsets	Middle to late stages (RPL22L1)	VEGF signaling
RPL34	Regulation of enzyme activity	Toll-like receptor signaling pathways	LY96	Herpes simplex virus 1 infection	ETS1	Neutrophils	Regulatory T cells	B cell subsets	middle stage (LY96)	
LY96	Mitochondrial respiratory chain complexes	Complications and associated pathways		Ribosome	YY1			NK cells		
RSL24D1	Toll-like receptor binding	Infectious diseases		Dilated cardiomyopathy	GATA3					
RPL22L1				Spliceosome	ZNK384					
RPS7				Cardiac muscle contraction						
COX7C				Protein export						
				Gap junction						
				Basal transcription factors						

^1^ Results shared between psoriatic arthritis and rheumatoid arthritis.

^2^ Results from rheumatoid arthritis scRNA-seq analysis.

## 4. Discussion

PsA and RA are two prevalent inflammatory rheumatic musculoskeletal diseases with overlapping clinical manifestations, immune-related joint pathological processes, and various systemic symptoms. Previous studies have primarily focused on elucidating the differences between PsA and RA concerning serum protein biomarkers, serum metabolomics, lipidomics, and fecal metabolomics [28–30]. In contrast, research aimed at identifying the similarities between these two diseases has been quite limited. The aim of this study was to identify common biomarkers and potential pathways between PsA and RA, thereby providing new insights into inflammatory rheumatic musculoskeletal diseases.

Our study identified seven genes that are upregulated in both PsA and RA. Enrichment analysis revealed significant associations with mitochondrial respiratory chain complex IV and Toll-like receptor binding. Mitochondrial respiratory complexes play a crucial role in the respiratory chain and are a major source of reactive oxygen species (ROS). In RA, the formation of pannus and disruption of the microvascular structure create a hypoxic environment in the synovium, leading to mitochondrial dysfunction and increased ROS production. This overproduction of ROS has been linked to cartilage and bone destruction in RA [31–33]. Elevated ROS levels are also observed in the serum, circulating cells, and joints of PsA patients, contributing to atherosclerosis, a common comorbidity in PsA [34–36]. Therefore, mitochondrial respiratory chain complex IV may be a key mediator of ROS increases due to hypoxia, resulting in inflammatory damage both intra- and extra-articularly.

Toll-like receptors (TLRs) are transmembrane proteins found on various immune and non-immune cells, including fibroblasts, chondrocytes, keratinocytes, and endothelial. These receptors are activated by damage-associated molecular patterns and pathogen-associated molecular patterns. Previous studies suggested that activated TLRs on both immune and non-immune cells can induce tissue damage in RA, perpetuating a cycle of inflammation through the release of endogenous TLR ligands [37]. Furthermore, TLR4 has been implicated in psoriasis and related conditions such as Crohn’s disease, ulcerative colitis, and atherosclerosis [38–40]. TLR2 overexpression on monocytes may mediate PsA joint damage induced by Gram-positive bacteria [41]. Collectively, this evidence underscores the unique role of Toll-like receptor-associated pathways in PsA and RA.

The KEGG analysis revealed pathways commonly shared between PsA and RA, specifically the NF-κB signaling pathway and the Toll-like receptor signaling pathway. NF-κB is a transcription factor that induces pro-inflammatory circuits and plays a critical role in the chronic inflammatory response associated with PsA [42]. In psoriatic synovium, NF-κB is implicated in the proliferation of osteoclast precursors and their differentiation into multinucleated osteoclasts [43]. Additionally, NF-κB triggers osteoclast generation and bone erosion in RA [44]. Furthermore, NF-κB activates genes linked to cardiovascular diseases, such as cardiac remodeling and heart failure, as well as metabolic disorders like type 2 diabetes mellitus and obesity—conditions that often complicate PsA and RA [45, 46]. Our findings suggest that the NF-κB signaling pathway may also be activated in the peripheral blood of PsA and RA patients, serving similar functions.

We finally identified 2 marker genes by the machine learning algorithm: RPL22L1 and LY96. Ribosomal protein RPL22L1 is a homologous analogue of RPL22 that plays pivotal roles in a variety of human cancers [47]. Lymphocyte antigen 96 (LY96), also known as myeloid differentiation 2 (MD2), plays a critical role in lipopolysaccharide (LPS)-induced inflammatory responses by serving as an accessory protein to Toll-like receptor 4 (TLR4) [48]. Both markers were upregulated in comparison to healthy samples. The diagnostic utility of these genes was confirmed through ROC and DCA analyses, and further validated using external datasets from blood, synovium, and skin. The consistent expression changes observed in synovium and skin suggest that RPL22L1 and LY96 contribute to disease mechanisms beyond joint pathology.

Through GSEA, we identified potential roles for these genes in arthritis-related processes, including osteoclast differentiation, autophagy, SNARE interactions in vesicular transport, and Fc gamma R-mediated phagocytosis. Notably, RPL22L1 and LY96 share a pathway related to gap junctions. Gap junctions, found in joint tissues, facilitate the transfer of signaling molecules between adjacent cells, promoting cellular interconnectivity [49]. The predominant connexin gene expressed is connexin 43, a protein found in synovial tissue and cells. Connexin 43, the primary connexin gene expressed in synovial tissue and cells, may play a pivotal role in RA pathophysiology by enhancing the expression of pro-inflammatory cytokines and chemokines [50]. Gap junctions are widely distributed across various cell types, including immune, endothelial, and stromal cells, suggesting their involvement in multiple pathological pathways of PsA and RA [51]. These findings imply that RPL22L1 and LY96 contribute to the pathogenesis of PsA and RA across multiple tissues, making them valuable biomarkers for disease detection in blood, synovium, and skin.

The study assessed immune cell infiltration in PsA and RA using CIBERSORT, providing evidence of immune system involvement in both diseases. Notably, no shared alterations in immune cell types were observed between PsA and RA, underscoring significant immunological differences between the two. Correlation analysis revealed a positive association between RPL22L1 and activated memory CD4 T cells in both PsA and RA, while a negative correlation was noted with neutrophils. Similarly, LY96 exhibited a negative correlation with CD8 T cells and regulatory T cells in both diseases. To further elucidate the mechanism at the cellular level, we performed scRNA-seq analysis. The results showed that RA patients exhibited significantly elevated proportions of memory CD8+ T cells, naïve CD4+ T cells and naïve CD8+ T cells. Furthermore, both RPL22L1 and LY96 were differentially expressed across T cell subsets, B cell subsets, and NK cells.

In both PsA and RA, CD4+ memory T cells migrate from peripheral blood to the synovial membrane, contributing to the inflammatory response [52]. The CD4/CD8 T cell ratio is notably lower in PsA than in RA, particularly within synovial fluid and the enthesis [53]. CD40L, a costimulatory molecule and early activation marker of T cells, is elevated in peripheral blood T cells of PsA patients compared to RA and healthy controls [54]. In PsA, the suppressive function of regulatory T cells (Tregs) on effector T cells is impaired. However, the role of Tregs in both PsA and RA remains debated, likely due to challenges in identifying these cells [55, 56]. Neutrophils regulate innate and adaptive immune responses in RA joints by endowing resident fibroblast-like synoviocytes with antigen-presenting cell capabilities and an inflammatory phenotype [57]. Consequently, RPL22L1 and LY96 may help regulate immune cells and contribute to the development of arthritis. Together with the GSEA findings, changes in gap junction could have an active role in regulating immune cells via these two genes.

Our pseudotime analysis showed that RPL22L1 was expressed in the middle and late stages, whereas LY96 was mainly expressed in the middle stages. The critical role of RPL22L1 in T cell development has been well-documented. Knockdown of RPL22L1 disrupts the emergence of hematopoietic stem cells in the aorta-gonad-mesonephros, primarily by inhibiting Smad1 expression and thereby preventing the induction of the key transcriptional regulator Runx1 [58, 59]. In contrast, the role of LY96 in T cell development remains unclear and warrants further investigation.

Analysis of cellular communication revealed that T cells communicated closely with NK cells and monocytes. Cellular communication was stronger in T cells with high expression of RPL22L1 and attenuated in T cells with high expression of LY96. Wang et al. proposed that a high percentage of CD38+ NK cells and a low percentage of CD38+ NK-like T cells in RA disrupt immune tolerance by stimulating mTOR signaling in CD4+ T cells and inhibiting the differentiation of regulatory T cells [60]. The communication between monocytes/macrophages and T cells is a complex process. Synovial monocytes/macrophages secrete inflammatory cytokines and chemokines that attract and balance CD4+ T cells in the synovium. Monocyte activation has been shown to influence the differentiation of Th1/Th17 cells from CD4+ T cells. Additionally, CD4+ effector T cells can activate, polarize, and kill monocytes/macrophages, as well as affect monocyte chemotaxis. In contrast, CD4+ Tregs promote monocyte/macrophage survival and induce anti-inflammatory monocytes/macrophages [61]. This complex interplay suggests that the communication networks influenced by RPL22L1 and LY96 may impact immune regulation in both PsA and RA.

The results of marker genes-associated cellular communication indicated that RPL22L1 and LY96 were involved in T cell communication, including signaling of vascular endothelial growth factor, CCL, IL2, LT, MIF, TGFb, PARs, FLT3, IL6, CD40, FASLG, ACTIVIN, and FGF. In addition, we observed enhanced VEGF signaling in both RPL22L1 and LY96 highly expressed T cells. Autocrine VEGF-mediated signaling coordinates lipid synthesis and mitochondrial function with T-cell activation to promote survival and immune responses [62]. RPL22L1 and LY96 are closely associated with enhanced VEGF signaling in T-cells from patients with PsA and RA, which may be one of the mechanisms by which T-cells are persistently activated and lead to inflammatory responses.

This study represents the first transcriptomic-level analysis to investigate common genes and mechanisms between PsA and RA. The approach to studying two diseases is valid as it has been successfully applied in various contexts [63, 64]. Our primary objective was to explore the mechanisms underlying arthropathy in both conditions. To identify DEGs linked to PsA, we compared healthy and psoriatic joint groups within the dataset, rather than psoriatic skin lesion versus joint groups. This method is essential due to the systemic, multi-organ nature of PsA and RA, where numerous genes are implicated in both joint and skin lesions. By maintaining this approach, we ensured that genes common to both tissues were preserved in our analysis.

The test set samples were derived from PBMCs, suggesting that the identified DEGs may be linked to extra-articular diseases and complications rather than arthropathy specifically. To validate the two marker genes, we aimed to use transcriptional data from the synovium. Despite exhaustive efforts, synovial transcriptome data for PsA patients was unavailable in online databases, so we opted to use a psoriatic skin dataset as a viable alternative. Interestingly, validating marker genes using synovial and skin tissues provides additional insights, revealing their role in the systemic and multiorgan pathogenesis. To identify marker genes, we attempted to use the protein interaction method based on DEGs, but this approach proved suboptimal due to the limited number of DEGs (**S2 Fig**). Given these constraints, employing machine learning to screen for marker genes was a reliable approach.

It is important to note that our use of public datasets lacks critical background information, such as patient demographics, disease stages, or treatment histories, potentially introducing bias into the results. Furthermore, our experimental validation involved a small sample size, limited to blood samples, and did not establish causality. Future studies should include larger sample sizes, encompass diverse tissues, and further explore causal relationships between identified genes and disease mechanisms.

## 5. Conclusion

This bioinformatics study employed machine learning approaches to identify RPL22L1 and LY96 as key marker genes shared by PsA and RA, with gap junctions playing a critical role in their contribution to disease development. Insights from immune cell infiltration and single-cell analyses have deepened our understanding of the involvement of RPL22L1 and LY96 in T cell differentiation and the activation of T cell VEGF signaling, which may be pivotal to the pathogenesis of these diseases.

In the future, RPL22L1 and LY96 may serve as potential biomarkers for diagnosing PsA, RA, and other inflammatory rheumatic musculoskeletal diseases. These biomarkers could be applied to various sample types, including blood and synovial tissue, thereby demonstrating significant clinical potential. Future research should investigate the relationship between these biomarkers and disease staging or severity. Moreover, comprehensive studies on RPL22L1 and LY96 are necessary to further elucidate their roles in the pathogenesis of PsA and RA and to explore potential therapeutic strategies. Future investigations may specifically target gap junction impairment and aberrant T-cell VEGF signaling associated with RPL22L1 and LY96.

## Supporting information

S1 Fig The Venn diagram shows intersection of GSEA results from RPL22L1 and LY96 in PsA and RA. (PDF)

S2 Fig Protein interaction based on DEGs. (PDF)

S1 Table Primer pairs for RT-qPCR. (PDF)

S2 Table GSEA results of markers in PsA and RA. (PDF)

S3 Table Common pathways affected by two core biomarkers. (PDF)
