# Supplementary figures and images for: The shared biomarkers and immune landscape in psoriatic arthritis and rheumatoid arthritis: Findings based on bioinformatics, machine learning and single-cell analysis
Source: PLoS One. 2024 Nov 7;19(11):e0313344. doi: 10.1371/journal.pone.0313344 (PMC11542839; doi:10.1371/journal.pone.0313344)

**S1 Fig**

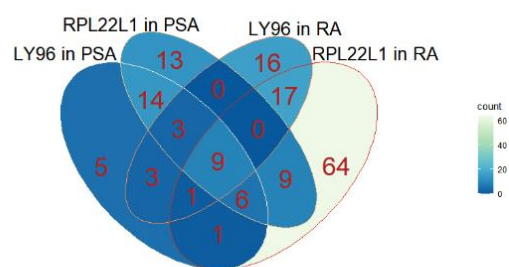

Supplement: S1 Fig — (PDF) [file pone.0313344.s001.pdf]

**S2 Fig**

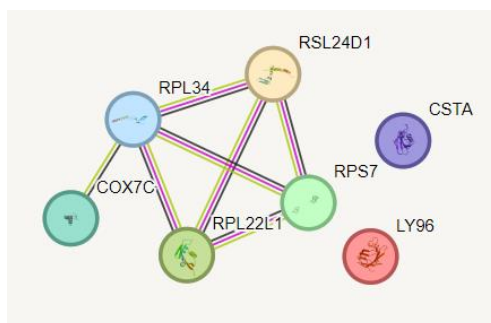

Supplement: S2 Fig — (PDF) [file pone.0313344.s002.pdf]
