# Supplementary material for: The shared biomarkers and immune landscape in psoriatic arthritis and rheumatoid arthritis: Findings based on bioinformatics, machine learning and single-cell analysis
Source: PLoS One. 2024 Nov 7;19(11):e0313344. doi: 10.1371/journal.pone.0313344 (PMC11542839; doi:10.1371/journal.pone.0313344)
Supplement: S1 Table — (PDF) [file pone.0313344.s003.pdf]

S1 Table

Gene	Forward primer (from 5' to 3')	Reverse primer (from 5' to 3')
RPL22L1	TGGAAATCTCGGGAATGTTGTTCAC	GCAACCACTCGAAGCCAATCAC
LY96	TGCCGAGGATCTGATGACGATTAC	CACCAATCTCTTCCCACCCA
GAPDH	CACCCACTCCTCCACCTTTGAC	GTCCACCACCCTGTTGCTGTAG
