# Supplementary material for: The shared biomarkers and immune landscape in psoriatic arthritis and rheumatoid arthritis: Findings based on bioinformatics, machine learning and single-cell analysis
Source: PLoS One. 2024 Nov 7;19(11):e0313344. doi: 10.1371/journal.pone.0313344 (PMC11542839; doi:10.1371/journal.pone.0313344)
Supplement: S2 Table — (PDF) [file pone.0313344.s004.pdf]

S2 Table

GSEA results of LY96 in PSA

ID	Description	setSize	enrichmentScore	NES	pvalue	p.adjust	qvalue	rank
hsa03060	Protein export	23	0.522330937	1.637472525	0.011299435	0.011299435	0.134037833	5729
hsa03010	Ribosome	131	0.517703784	2.32576182	0.001862197	0.001862197	0.052604648	3013
hsa04130	SNARE interactions in vesicular transport	32	0.450208806	1.522127747	0.027573529	0.027573529	0.255884721	5541
hsa04136	Autophagy - other	31	0.439786623	1.481516852	0.038251366	0.038251366	0.310461226	5367
hsa05168	Herpes simplex virus 1 infection	473	0.421132353	2.191932684	0.001818182	0.001818182	0.052604648	4177
hsa05143	African trypanosomiasis	36	0.420827531	1.468650292	0.040366972	0.040366972	0.319231388	3210
hsa03022	Basal transcription factors	41	0.417935067	1.502348943	0.029038113	0.029038113	0.255884721	4989
hsa05134	Legionellosis	56	0.404688335	1.535039	0.015267176	0.015267176	0.168168513	5024
hsa03460	Fanconi anemia pathway	52	0.389829405	1.447450845	0.034351145	0.034351145	0.287438073	5960
hsa05171	Coronavirus disease - COVID-19	222	0.368028803	1.790003194	0.001808318	0.001808318	0.052604648	3059
hsa03040	Spliceosome	128	0.351727988	1.569176781	0.005597015	0.005597015	0.101543367	6074
hsa04140	Autophagy - animal	139	0.322585625	1.455124227	0.016885553	0.016885553	0.179581385	5541
hsa04620	Toll-like receptor signaling pathway	95	0.316608776	1.341873762	0.04494382	0.04494382	0.33003858	3956
hsa04120	Ubiquitin mediated proteolysis	141	0.298092767	1.348757054	0.041431262	0.041431262	0.319456834	4936
hsa04510	Focal adhesion	201	-0.269053846	-1.30188402	0.02832244	0.02832244	0.255884721	5571
hsa04530	Tight junction	164	-0.277870274	-1.310786316	0.034482759	0.034482759	0.287438073	3854
hsa04310	Wnt signaling pathway	165	-0.301495368	-1.421493838	0.004347826	0.004347826	0.083810069	4403
hsa04270	Vascular smooth muscle contraction	128	-0.307745048	-1.403044325	0.008583691	0.008583691	0.125450906	4947
hsa04514	Cell adhesion molecules	151	-0.322041391	-1.490362053	0.002192982	0.002192982	0.052604648	5112
hsa04261	Adrenergic signaling in cardiomyocytes	153	-0.326082782	-1.511362396	0.002159827	0.002159827	0.052604648	4996
hsa04670	Leukocyte transendothelial migration	110	-0.333432023	-1.491288147	0.006369427	0.006369427	0.103833819	4976
hsa04727	GABAergic synapse	89	-0.335520907	-1.430126229	0.023255814	0.023255814	0.224143207	3244
hsa04970	Salivary secretion	87	-0.336119715	-1.432620782	0.020920502	0.020920502	0.208139461	4947
hsa04611	Platelet activation	121	-0.341203988	-1.546521003	0.002079002	0.002079002	0.052604648	4813
hsa04927	Cortisol synthesis and secretion	65	-0.343921015	-1.398073593	0.043572985	0.043572985	0.32777624	3244
hsa04024	cAMP signaling pathway	224	-0.347943471	-1.714492809	0.002202643	0.002202643	0.052604648	4869
hsa04540	Gap junction	87	-0.349405865	-1.489249457	0.012552301	0.012552301	0.143384962	4779
hsa04911	Insulin secretion	85	-0.355895949	-1.504781173	0.010615711	0.010615711	0.130964354	3244
hsa05412	Arrhythmogenic right ventricular cardiomyopathy	77	-0.357479593	-1.500962616	0.010526316	0.010526316	0.130964354	5952
hsa04020	Calcium signaling pathway	238	-0.358572752	-1.774935544	0.002217295	0.002217295	0.052604648	4842
hsa04972	Pancreatic secretion	96	-0.359413956	-1.555825566	0.006396588	0.006396588	0.103833819	4767
hsa04260	Cardiac muscle contraction	85	-0.361307493	-1.527661986	0.010615711	0.010615711	0.130964354	4633
hsa05032	Morphine addiction	91	-0.369726511	-1.577466773	0.004338395	0.004338395	0.083810069	3244
hsa05416	Viral myocarditis	59	-0.371794679	-1.475736013	0.019736842	0.019736842	0.202908587	5866
hsa04080	Neuroactive ligand-receptor interaction	355	-0.387845536	-2.012191857	0.002136752	0.002136752	0.052604648	3795
hsa05150	Staphylococcus aureus infection	87	-0.39551859	-1.685792668	0.0041841	0.0041841	0.083810069	5590
hsa05410	Hypertrophic cardiomyopathy	90	-0.398331734	-1.697929983	0.002141328	0.002141328	0.052604648	4243
hsa05414	Dilated cardiomyopathy	95	-0.404393838	-1.750734512	0.002136752	0.002136752	0.052604648	4243
hsa04742	Taste transduction	83	-0.452285971	-1.915253678	0.002096436	0.002096436	0.052604648	1830

hsa04975	Fat digestion and absorption	42	-0.482742734	-1.80156796	0.008830022	0.008830022	0.125450906	3533
hsa05033	Nicotine addiction	40	-0.491508799	-1.796941821	0.008948546	0.008948546	0.125450906	3110
hsa04740	Olfactory transduction	152	-0.491795032	-2.275956749	0.002173913	0.002173913	0.052604648	3244

GSEA results of RPL22L1 in PSA

ID	Description	setSize	enrichmentScore	NES	pvalue	p.adjust	qvalue	rank
hsa03060	Protein export	23	0.590000699	1.928343377	0.002118644	0.002118644	0.054293222	4730
hsa03010	Ribosome	131	0.566238932	2.684260349	0.002132196	0.002132196	0.054293222	3151
hsa03022	Basal transcription factors	41	0.433504991	1.672953234	0.006396588	0.006396588	0.126136236	4483
hsa04136	Autophagy - other	31	0.416682404	1.509872038	0.036437247	0.036437247	0.239505647	5021
hsa05168	Herpes simplex virus 1 infection	473	0.410683352	2.27138758	0.002202643	0.002202643	0.054293222	4477
hsa04130	SNARE interactions in vesicular transport	32	0.405578195	1.469772694	0.040899796	0.040899796	0.251046195	5641
hsa03460	Fanconi anemia pathway	52	0.376112824	1.542404325	0.029350105	0.029350105	0.222601335	4221
hsa05171	Coronavirus disease - COVID-19	222	0.375606048	1.920840411	0.002096436	0.002096436	0.054293222	3142
hsa03040	Spliceosome	128	0.371340286	1.761869584	0.002118644	0.002118644	0.054293222	6394
hsa05134	Legionellosis	56	0.344348437	1.421174175	0.045652174	0.045652174	0.259681394	4621
hsa03013	Nucleocytoplasmic transport	102	0.31516967	1.44307892	0.023861171	0.023861171	0.210066275	5649
hsa03015	mRNA surveillance pathway	91	0.30137048	1.354829881	0.048140044	0.048140044	0.263691078	5624
hsa05165	Human papillomavirus infection	324	-0.254175662	-1.336243273	0.007285974	0.007285974	0.126771445	5813
hsa04010	MAPK signaling pathway	299	-0.255896982	-1.335616554	0.014787431	0.014787431	0.166302325	5558
hsa04015	Rap1 signaling pathway	210	-0.258953692	-1.289567432	0.041509434	0.041509434	0.251046195	4957
hsa04814	Motor proteins	189	-0.269698101	-1.330199012	0.028248588	0.028248588	0.222601335	5123
hsa04530	Tight junction	164	-0.276777964	-1.337669553	0.031835206	0.031835206	0.231841996	6114
hsa04934	Cushing syndrome	155	-0.281063464	-1.345214141	0.034285714	0.034285714	0.239505647	3780
hsa04390	Hippo signaling pathway	155	-0.288089106	-1.378839972	0.020952381	0.020952381	0.206583124	6188
hsa04510	Focal adhesion	201	-0.289107262	-1.433870717	0.011450382	0.011450382	0.141120932	5696
hsa04611	Platelet activation	121	-0.28953377	-1.335728016	0.044692737	0.044692737	0.259208653	5729
hsa04919	Thyroid hormone signaling pathway	121	-0.290311255	-1.339314846	0.04283054	0.04283054	0.253376458	4737
hsa04360	Axon guidance	181	-0.293194428	-1.437785093	0.011278195	0.011278195	0.141120932	6323
hsa04261	Adrenergic signaling in cardiomyocytes	153	-0.29555866	-1.412544024	0.015180266	0.015180266	0.166302325	4569
hsa04928	Parathyroid hormone synthesis, secretion and action	106	-0.299109678	-1.353005028	0.038745387	0.038745387	0.249140821	4797
hsa05225	Hepatocellular carcinoma	166	-0.304092328	-1.468798744	0.013232514	0.013232514	0.156561536	5826
hsa04933	AGE-RAGE signaling pathway in diabetic complications	100	-0.308895156	-1.382357276	0.035250464	0.035250464	0.239505647	6436
hsa04911	Insulin secretion	85	-0.310970512	-1.359982886	0.04676259	0.04676259	0.260978903	4542
hsa04514	Cell adhesion molecules	151	-0.31439568	-1.499053832	0.011406844	0.011406844	0.141120932	6494
hsa04270	Vascular smooth muscle contraction	128	-0.318942745	-1.474274126	0.009433962	0.009433962	0.139523337	4700
hsa04020	Calcium signaling pathway	238	-0.323288342	-1.639006418	0.001904762	0.001904762	0.054293222	4885
hsa04024	cAMP signaling pathway	224	-0.324207295	-1.627670344	0.001876173	0.001876173	0.054293222	4737
hsa04540	Gap junction	87	-0.326583284	-1.428	0.029250457	0.029250457	0.222601335	5137
hsa04260	Cardiac muscle contraction	85	-0.329550881	-1.441241342	0.026978417	0.026978417	0.221664773	4195
hsa04721	Synaptic vesicle cycle	78	-0.332815608	-1.421912723	0.036297641	0.036297641	0.239505647	2283
hsa04610	Complement and coagulation cascades	85	-0.336109973	-1.469926547	0.023381295	0.023381295	0.210066275	4278

hsa04972	Pancreatic secretion	96	-0.344610201	-1.526213261	0.009328358	0.009328358	0.139523337	4281
hsa04742	Taste transduction	83	-0.344679897	-1.492756152	0.02037037	0.02037037	0.206583124	3153
hsa04730	Long-term depression	58	-0.346361306	-1.398471714	0.041587902	0.041587902	0.251046195	4885
hsa04927	Cortisol synthesis and secretion	65	-0.347613158	-1.449258952	0.023941068	0.023941068	0.210066275	4091
hsa04330	Notch signaling pathway	58	-0.354475292	-1.431232821	0.032136106	0.032136106	0.231841996	3195
hsa05414	Dilated cardiomyopathy	95	-0.374166513	-1.656343685	0.005514706	0.005514706	0.117159813	4538
hsa04080	Neuroactive ligand-receptor interaction	355	-0.38014939	-2.018200838	0.001845018	0.001845018	0.054293222	3423
hsa05412	Arrhythmogenic right ventricular cardiomyopathy	77	-0.386057734	-1.646454626	0.007168459	0.007168459	0.126771445	4538
hsa05217	Basal cell carcinoma	63	-0.386449354	-1.596801431	0.005545287	0.005545287	0.117159813	6539
hsa04370	VEGF signaling pathway	57	-0.389004358	-1.562573896	0.009328358	0.009328358	0.139523337	5320
hsa05410	Hypertrophic cardiomyopathy	90	-0.406327397	-1.784451307	0.001841621	0.001841621	0.054293222	3467
hsa05033	Nicotine addiction	40	-0.41076395	-1.539954177	0.016791045	0.016791045	0.177379082	3705
hsa05216	Thyroid cancer	37	-0.41686681	-1.52876378	0.024528302	0.024528302	0.210066275	4675
hsa05150	Staphylococcus aureus infection	87	-0.417675394	-1.826304322	0.001828154	0.001828154	0.054293222	6497
hsa05219	Bladder cancer	41	-0.420083645	-1.581704286	0.011257036	0.011257036	0.141120932	4805
hsa05416	Viral myocarditis	59	-0.456445979	-1.844374718	0.001904762	0.001904762	0.054293222	6494
hsa04740	Olfactory transduction	152	-0.47461875	-2.266263226	0.001883239	0.001883239	0.054293222	4381
hsa04950	Maturity onset diabetes of the young	26	-0.484661398	-1.629790797	0.024856597	0.024856597	0.210066275	4349

GSEA results of LY96 in RA

ID	Description	setSize	enrichmentScore	NES	pvalue	p.adjust	qvalue	rank
hsa03010	Ribosome	112	0.750597942	2.271579427	0.00011152	0.00011152	0.006714679	2456
hsa03060	Protein export	23	0.674377437	1.631611145	0.009818077	0.009818077	0.140750374	1909
hsa00900	Terpenoid backbone biosynthesis	22	0.671800065	1.608150649	0.011958582	0.011958582	0.147264879	3342
hsa04130	SNARE interactions in vesicular transport	33	0.591566902	1.524294054	0.022510232	0.022510232	0.206158713	4740
hsa03022	Basal transcription factors	40	0.589589743	1.565574888	0.012229164	0.012229164	0.147264879	4198
hsa05171	Coronavirus disease - COVID-19	205	0.58934648	1.860909297	0.00010445	0.00010445	0.006714679	2641
hsa05134	Legionellosis	53	0.588627998	1.630785877	0.004277806	0.004277806	0.080490292	4260
hsa00190	Oxidative phosphorylation	120	0.583817107	1.775604422	0.000110497	0.000110497	0.006714679	2651
hsa05143	African trypanosomiasis	34	0.555633937	1.438612148	0.048886475	0.048886475	0.2920428	2005
hsa04932	Non-alcoholic fatty liver disease	147	0.517839021	1.601153774	0.000324254	0.000324254	0.013945369	3375
hsa04714	Thermogenesis	215	0.516333139	1.635118093	0.000104069	0.000104069	0.006714679	2815
hsa04621	NOD-like receptor signaling pathway	173	0.514654168	1.609069722	0.000319285	0.000319285	0.013945369	2787
hsa04620	Toll-like receptor signaling pathway	95	0.503038618	1.495716495	0.009265614	0.009265614	0.139471878	3855
hsa04657	IL-17 signaling pathway	92	0.500769177	1.484828041	0.011461318	0.011461318	0.147264879	2783
hsa05415	Diabetic cardiomyopathy	188	0.495419709	1.557176962	0.000631978	0.000631978	0.023782333	2651
hsa04260	Cardiac muscle contraction	82	0.492170813	1.438455369	0.021535581	0.021535581	0.206158713	2584
hsa05168	Herpes simplex virus 1 infection	456	0.488777662	1.587241554	0.000100513	0.000100513	0.006714679	4279
hsa04668	TNF signaling pathway	111	0.487251334	1.473759857	0.008595669	0.008595669	0.139471878	4486
hsa04622	RIG-I-like receptor signaling pathway	69	0.483350647	1.384214333	0.047458036	0.047458036	0.2920428	2804
hsa04623	Cytosolic DNA-sensing pathway	70	0.482945895	1.385329855	0.045607702	0.045607702	0.2920428	3431
hsa03040	Spliceosome	119	0.475499762	1.445779714	0.0116189	0.0116189	0.147264879	3929

hsa03018	RNA degradation	75	0.473887103	1.369284552	0.048885179	0.048885179	0.2920428	2730
hsa05208	Chemical carcinogenesis - reactive oxygen species	206	0.472750426	1.492990704	0.001462599	0.001462599	0.044031935	2815
hsa05417	Lipid and atherosclerosis	203	0.471588098	1.488144757	0.001255493	0.001255493	0.041996601	3880
hsa04723	Retrograde endocannabinoid signaling	138	0.467948583	1.438790993	0.009264305	0.009264305	0.139471878	2305
hsa04140	Autophagy - animal	140	0.449981355	1.385483225	0.018156121	0.018156121	0.195212428	4024
hsa05012	Parkinson disease	243	0.447671914	1.425860857	0.002067397	0.002067397	0.047876566	2710
hsa04120	Ubiquitin mediated proteolysis	137	0.445073693	1.367974055	0.023967752	0.023967752	0.206158713	4192
hsa05020	Prion disease	251	0.440728897	1.406135861	0.002989691	0.002989691	0.06428959	2689
hsa05014	Amyotrophic lateral sclerosis	338	0.431287821	1.390212981	0.002030457	0.002030457	0.047876566	2774
hsa05016	Huntington disease	283	0.430859232	1.382544255	0.003785554	0.003785554	0.075976723	2689
hsa04936	Alcoholic liver disease	135	0.42900798	1.316590922	0.047093531	0.047093531	0.2920428	3804
hsa05010	Alzheimer disease	356	0.415626774	1.34217576	0.005364372	0.005364372	0.094997556	4094
hsa05132	Salmonella infection	242	0.398847739	1.269720511	0.043127521	0.043127521	0.2920428	3682
hsa05022	Pathways of neurodegeneration - multiple diseases	444	0.389170733	1.263297569	0.016497334	0.016497334	0.183946885	2783
hsa04142	Lysosome	127	-0.327398973	-1.320095267	0.023890785	0.023890785	0.206158713	3075
hsa01200	Carbon metabolism	111	-0.336732613	-1.334552708	0.022030651	0.022030651	0.206158713	2999
hsa04640	Hematopoietic cell lineage	93	-0.338423917	-1.305091423	0.040593286	0.040593286	0.285245512	3179
hsa04658	Th1 and Th2 cell differentiation	89	-0.33903923	-1.299706797	0.040560472	0.040560472	0.285245512	4117
hsa05414	Dilated cardiomyopathy	93	-0.345411373	-1.332037711	0.036690086	0.036690086	0.285245512	3050
hsa04512	ECM-receptor interaction	86	-0.356506559	-1.354816555	0.029202279	0.029202279	0.244206194	3050
hsa04540	Gap junction	84	-0.365050709	-1.381104158	0.023743017	0.023743017	0.206158713	4373
hsa04612	Antigen processing and presentation	66	-0.365316959	-1.331014556	0.039840637	0.039840637	0.285245512	1935
hsa05412	Arrhythmogenic right ventricular cardiomyopathy	75	-0.387479574	-1.447936472	0.013003096	0.013003096	0.150562164	2581
hsa00970	Aminoacyl-tRNA biosynthesis	40	-0.435363094	-1.436370642	0.040742235	0.040742235	0.285245512	3985
hsa01230	Biosynthesis of amino acids	68	-0.461226498	-1.688071503	0.001732102	0.001732102	0.047404886	2995
hsa05340	Primary immunodeficiency	36	-0.465398227	-1.498871717	0.031502113	0.031502113	0.256318757	3033
hsa00860	Porphyrin metabolism	30	-0.47617308	-1.462925348	0.039507068	0.039507068	0.285245512	4279
hsa00515	Mannose type O-glycan biosynthesis	22	-0.562676279	-1.601927741	0.019077901	0.019077901	0.198050084	6313

GSEA results of RPL22L1 in RA

ID	Description	setSize	enrichmentScore	NES	pvalue	p.adjust	qvalue	rank
hsa03010	Ribosome	112	0.795724894	2.326962577	0.001072961	0.001072961	0.024557114	2981
hsa03060	Protein export	23	0.720931849	1.737814008	0.002677376	0.002677376	0.037964034	1152
hsa00900	Terpenoid backbone biosynthesis	22	0.704224407	1.684383559	0.004054054	0.004054054	0.051433705	3282
hsa03022	Basal transcription factors	40	0.636581173	1.665799271	0.001222494	0.001222494	0.024557114	3627
hsa03008	Ribosome biogenesis in eukaryotes	70	0.628152886	1.747527674	0.001149425	0.001149425	0.024557114	4015
hsa00190	Oxidative phosphorylation	120	0.615164473	1.819949973	0.001059322	0.001059322	0.024557114	3275
hsa03050	Proteasome	43	0.611157638	1.615522831	0.004854369	0.004854369	0.05850792	3012
hsa03018	RNA degradation	75	0.588856932	1.658279698	0.001136364	0.001136364	0.024557114	4977
hsa03040	Spliceosome	119	0.58017769	1.711532603	0.001062699	0.001062699	0.024557114	4261
hsa03440	Homologous recombination	41	0.574759911	1.505804839	0.028083028	0.028083028	0.085804212	4652
hsa03030	DNA replication	36	0.572989444	1.478404808	0.040740741	0.040740741	0.107919371	5140

hsa03460	Fanconi anemia pathway	48	0.562910697	1.509386961	0.017921147	0.017921147	0.081162502	3963
hsa05168	Herpes simplex virus 1 infection	456	0.552786978	1.710307481	0.000999001	0.000999001	0.024557114	4496
hsa05171	Coronavirus disease - COVID-19	205	0.552587669	1.683247364	0.001013171	0.001013171	0.024557114	3611
hsa03420	Nucleotide excision repair	57	0.543710681	1.487554834	0.015294118	0.015294118	0.080270607	3977
hsa04714	Thermogenesis	215	0.541149909	1.650316425	0.001010101	0.001010101	0.024557114	2856
hsa00280	Valine, leucine and isoleucine degradation	48	0.537136947	1.440277309	0.035842294	0.035842294	0.097077295	3245
hsa03013	Nucleocytoplasmic transport	99	0.529404488	1.530237623	0.002202643	0.002202643	0.033184558	3772
hsa01240	Biosynthesis of cofactors	135	0.522997823	1.551721706	0.00210084	0.00210084	0.033184558	4160
hsa05012	Parkinson disease	243	0.516620591	1.581643845	0.001008065	0.001008065	0.024557114	3091
hsa04932	Non-alcoholic fatty liver disease	147	0.508573276	1.521727993	0.002063983	0.002063983	0.033184558	3715
hsa04623	Cytosolic DNA-sensing pathway	70	0.507794737	1.412690088	0.026436782	0.026436782	0.085804212	1913
hsa04260	Cardiac muscle contraction	82	0.500876585	1.432208805	0.020022247	0.020022247	0.084520558	3523
hsa05020	Prion disease	251	0.481765234	1.478531069	0.00201005	0.00201005	0.033184558	3102
hsa05014	Amyotrophic lateral sclerosis	338	0.48017991	1.482007524	0.001001001	0.001001001	0.024557114	3122
hsa04110	Cell cycle	155	0.476518718	1.4287419	0.010341262	0.010341262	0.078467654	4340
hsa05016	Huntington disease	283	0.476043056	1.46454859	0.001006036	0.001006036	0.024557114	3054
hsa05415	Diabetic cardiomyopathy	188	0.468977487	1.422634991	0.007121058	0.007121058	0.077909706	2910
hsa04120	Ubiquitin mediated proteolysis	137	0.460251796	1.367002203	0.029288703	0.029288703	0.085804212	4586
hsa05208	Chemical carcinogenesis - reactive oxygen species	206	0.452949938	1.379845831	0.00810537	0.00810537	0.077909706	4337
hsa05010	Alzheimer disease	356	0.423786663	1.310057611	0.003996004	0.003996004	0.051433705	3107
hsa05022	Pathways of neurodegeneration - multiple diseases	444	0.395991106	1.224044825	0.016983017	0.016983017	0.080270607	3107
hsa05224	Breast cancer	143	-0.290237866	-1.228525858	0.046511628	0.046511628	0.108289592	4716
hsa05207	Chemical carcinogenesis - receptor activation	180	-0.291717759	-1.222380964	0.047619048	0.047619048	0.108289592	2727
hsa05160	Hepatitis C	150	-0.295099772	-1.229665557	0.027777778	0.027777778	0.085804212	3106
hsa05206	MicroRNAs in cancer	162	-0.295185623	-1.257967325	0.028571429	0.028571429	0.085804212	4961
hsa05202	Transcriptional misregulation in cancer	175	-0.296609949	-1.266189693	0.047619048	0.047619048	0.108289592	1729
hsa04152	AMPK signaling pathway	118	-0.302719772	-1.261466877	0.029850746	0.029850746	0.085804212	4102
hsa04071	Sphingolipid signaling pathway	117	-0.308404887	-1.278118041	0.029411765	0.029411765	0.085804212	2706
hsa04910	Insulin signaling pathway	134	-0.308969509	-1.307080101	0.045454545	0.045454545	0.108289592	4102
hsa04740	Olfactory transduction	120	-0.314620538	-1.31615567	0.034482759	0.034482759	0.09445636	8371
hsa04022	cGMP-PKG signaling pathway	162	-0.315571996	-1.344846189	0.028571429	0.028571429	0.085804212	4194
hsa05146	Amoebiasis	101	-0.316645769	-1.310209321	0.047058824	0.047058824	0.108289592	1874
hsa05414	Dilated cardiomyopathy	93	-0.316904145	-1.272824716	0.042553191	0.042553191	0.108289592	4194
hsa05161	Hepatitis B	158	-0.31821715	-1.340233831	0.029411765	0.029411765	0.085804212	3692
hsa04935	Growth hormone synthesis, secretion and action	118	-0.318332076	-1.326525079	0.029850746	0.029850746	0.085804212	4752
hsa04530	Tight junction	159	-0.324431411	-1.378840984	0.029411765	0.029411765	0.085804212	3609
hsa04012	ErbB signaling pathway	84	-0.334696842	-1.33392833	0.029126214	0.029126214	0.085804212	2204
hsa04066	HIF-1 signaling pathway	107	-0.334944972	-1.370792215	0.028571429	0.028571429	0.085804212	3378
hsa04520	Adherens junction	90	-0.337106823	-1.330813545	0.030612245	0.030612245	0.085804212	3609
hsa04360	Axon guidance	180	-0.343195347	-1.438086802	0.047619048	0.047619048	0.108289592	3974
hsa05152	Tuberculosis	170	-0.343953898	-1.463542404	0.038461538	0.038461538	0.103013945	2397
hsa05203	Viral carcinogenesis	184	-0.345020269	-1.461521957	0.047619048	0.047619048	0.108289592	1718
hsa05150	Staphylococcus aureus infection	76	-0.345354834	-1.339809471	0.042016807	0.042016807	0.108289592	1346

hsa04270	Vascular smooth muscle contraction	126	-0.347007682	-1.48768697	0.016949153	0.016949153	0.080270607	4194
hsa05214	Glioma	73	-0.347539642	-1.348163149	0.045801527	0.045801527	0.108289592	3206
hsa04640	Hematopoietic cell lineage	93	-0.352264276	-1.414846365	0.021276596	0.021276596	0.084520558	2374
hsa04650	Natural killer cell mediated cytotoxicity	121	-0.35686119	-1.499451228	0.017857143	0.017857143	0.081162502	2680
hsa04933	AGE-RAGE signaling pathway in diabetic complications	99	-0.357405817	-1.474892865	0.021276596	0.021276596	0.084520558	2706
hsa05135	Yersinia infection	133	-0.362751739	-1.535292552	0.02173913	0.02173913	0.084520558	3704
hsa01521	EGFR tyrosine kinase inhibitor resistance	78	-0.363630422	-1.416141382	0.026548673	0.026548673	0.085804212	2118
hsa01522	Endocrine resistance	95	-0.363816796	-1.470855381	0.020408163	0.020408163	0.084520558	3529
hsa05140	Leishmaniasis	72	-0.366324498	-1.418276453	0.023076923	0.023076923	0.085804212	4611
hsa04061	Viral protein interaction with cytokine and cytokine receptor	92	-0.367291148	-1.464236021	0.03125	0.03125	0.086584997	776
hsa04926	Relaxin signaling pathway	127	-0.36979965	-1.573714375	0.018518519	0.018518519	0.081162502	2706
hsa04917	Prolactin signaling pathway	69	-0.371113524	-1.421434888	0.014492754	0.014492754	0.080270607	2118
hsa05130	Pathogenic Escherichia coli infection	187	-0.371891619	-1.588384117	0.05	0.05	0.112641417	3663
hsa04512	ECM-receptor interaction	86	-0.371996756	-1.45879432	0.03030303	0.03030303	0.085804212	767
hsa05215	Prostate cancer	96	-0.3720017	-1.51106885	0.020833333	0.020833333	0.084520558	3268
hsa04630	JAK-STAT signaling pathway	161	-0.376563795	-1.604427142	0.03030303	0.03030303	0.085804212	2944
hsa05418	Fluid shear stress and atherosclerosis	132	-0.376564645	-1.588898356	0.02173913	0.02173913	0.084520558	2204
hsa04931	Insulin resistance	107	-0.379839899	-1.554528716	0.014285714	0.014285714	0.080270607	4102
hsa04072	Phospholipase D signaling pathway	143	-0.380928697	-1.6124042	0.023255814	0.023255814	0.085804212	2706
hsa04923	Regulation of lipolysis in adipocytes	56	-0.385936999	-1.398924703	0.046357616	0.046357616	0.108289592	2795
hsa04920	Adipocytokine signaling pathway	68	-0.388929414	-1.49363061	0.014388489	0.014388489	0.080270607	2936
hsa04722	Neurotrophin signaling pathway	116	-0.391583911	-1.625041814	0.014925373	0.014925373	0.080270607	3652
hsa05120	Epithelial cell signaling in Helicobacter pylori infection	69	-0.395132171	-1.513430842	0.014492754	0.014492754	0.080270607	1371
hsa05212	Pancreatic cancer	76	-0.396410576	-1.53788102	0.016806723	0.016806723	0.080270607	2159
hsa04370	VEGF signaling pathway	56	-0.396550696	-1.437396689	0.046357616	0.046357616	0.108289592	2159
hsa04145	Phagosome	139	-0.399257189	-1.675862523	0.023255814	0.023255814	0.085804212	3705
hsa00590	Arachidonic acid metabolism	55	-0.402542925	-1.446604194	0.047297297	0.047297297	0.108289592	1911
hsa04662	B cell receptor signaling pathway	82	-0.40477331	-1.586312532	0.009708738	0.009708738	0.078467654	2159
hsa05221	Acute myeloid leukemia	66	-0.4054042	-1.546122522	0.014492754	0.014492754	0.080270607	2341
hsa05231	Choline metabolism in cancer	95	-0.406116957	-1.641868431	0.010204082	0.010204082	0.078467654	2680
hsa04062	Chemokine signaling pathway	184	-0.408813835	-1.731754479	0.047619048	0.047619048	0.108289592	2706
hsa00561	Glycerolipid metabolism	62	-0.412657027	-1.533706996	0.013888889	0.013888889	0.080270607	3674
hsa04142	Lysosome	127	-0.416694344	-1.773278799	0.018518519	0.018518519	0.081162502	3216
hsa04721	Synaptic vesicle cycle	75	-0.418378336	-1.630878671	0.016393443	0.016393443	0.080270607	3911
hsa05213	Endometrial cancer	58	-0.423974124	-1.549862723	0.013513514	0.013513514	0.080270607	4582
hsa04928	Parathyroid hormone synthesis, secretion and action	104	-0.425428102	-1.747420134	0.013333333	0.013333333	0.080270607	4208
hsa05223	Non-small cell lung cancer	72	-0.43300819	-1.676451678	0.007692308	0.007692308	0.077909706	4752
hsa05100	Bacterial invasion of epithelial cells	76	-0.440705128	-1.709722424	0.008403361	0.008403361	0.077909706	2664
hsa04540	Gap junction	84	-0.441814182	-1.760842589	0.009708738	0.009708738	0.078467654	2706
hsa05220	Chronic myeloid leukemia	75	-0.448818851	-1.749538704	0.008196721	0.008196721	0.077909706	3001
hsa04610	Complement and coagulation cascades	82	-0.449883118	-1.76309853	0.009708738	0.009708738	0.078467654	1973
hsa04611	Platelet activation	119	-0.451287453	-1.885766142	0.016393443	0.016393443	0.080270607	2374
hsa05219	Bladder cancer	41	-0.4537443	-1.546626479	0.027322404	0.027322404	0.085804212	3529

hsa04670	Leukocyte transendothelial migration	108	-0.454055749	-1.871526755	0.014492754	0.014492754	0.080270607	3609
hsa04613	Neutrophil extracellular trap formation	151	-0.457435661	-1.906143818	0.026315789	0.026315789	0.085804212	2159
hsa04664	Fc epsilon RI signaling pathway	63	-0.459360453	-1.734601018	0.006666667	0.006666667	0.076524645	2431
hsa04330	Notch signaling pathway	57	-0.477278692	-1.73784961	0.013157895	0.013157895	0.080270607	3598
hsa04966	Collecting duct acid secretion	26	-0.492601688	-1.564847816	0.02811245	0.02811245	0.085804212	1371
hsa00410	beta-Alanine metabolism	31	-0.494635538	-1.581000012	0.023696682	0.023696682	0.085804212	2038
hsa04666	Fc gamma R-mediated phagocytosis	92	-0.503293457	-2.006420285	0.010416667	0.010416667	0.078467654	2159
hsa00531	Glycosaminoglycan degradation	18	-0.515164861	-1.447351048	0.0433213	0.0433213	0.108289592	7969
hsa04380	Osteoclast differentiation	126	-0.515916425	-2.211830409	0.016949153	0.016949153	0.080270607	4001
hsa04614	Renin-angiotensin system	22	-0.605139927	-1.79014387	0.011450382	0.011450382	0.080270607	1708
