# Supplementary material for: The shared biomarkers and immune landscape in psoriatic arthritis and rheumatoid arthritis: Findings based on bioinformatics, machine learning and single-cell analysis
Source: PLoS One. 2024 Nov 7;19(11):e0313344. doi: 10.1371/journal.pone.0313344 (PMC11542839; doi:10.1371/journal.pone.0313344)
Supplement: S3 Table — (PDF) [file pone.0313344.s005.pdf]

S3 Table

ID	Description
hsa05171	Coronavirus disease - COVID-19
hsa05168	Herpes simplex virus 1 infection
hsa03010	Ribosome
hsa05414	Dilated cardiomyopathy
hsa03040	Spliceosome
hsa04260	Cardiac muscle contraction
hsa03060	Protein export
hsa04540	Gap junction
hsa03022	Basal transcription factors
